# Engineering plant architecture via CRISPR/Cas9-mediated alteration of strigolactone biosynthesis

**DOI:** 10.1186/s12870-018-1387-1

**Published:** 2018-08-29

**Authors:** Haroon Butt, Muhammad Jamil, Jian You Wang, Salim Al-Babili, Magdy Mahfouz

**Affiliations:** 1 Laboratory for Genome Engineering, Biological and Environmental Sciences and Engineering Division, King Abdullah University of Science and Technology, Thuwal, 23955-6900 Saudi Arabia; 2 The Bioactives Lab, Biological and Environmental Sciences and Engineering Division, King Abdullah University of Science and Technology, Thuwal, 23955-6900 Saudi Arabia

**Keywords:** Genome editing, CRISPR/Cas9, Strigolactones, Plant architecture, Carotenoids, Carotenoid cleavage dioxygenases, CCD7, Crop improvement, Rice engineering

## Abstract

**Background:**

Precision plant genome engineering holds much promise for targeted improvement of crop traits via unprecedented single-base level control over the genetic material. Strigolactones (SLs) are a key determinant of plant architecture, known for their role in inhibiting shoot branching (tillering).

**Results:**

We used CRISPR/Cas9 in rice (*Oryza sativa*) for targeted disruption of *CAROTENOID CLEAVAGE DIOXYGENASE 7* (*CCD7*), which controls a key step in SL biosynthesis. The *ccd7* mutants exhibited a striking increase in tillering, combined with a reduced height, which could be rescued by application of the synthetic SL analog GR24. *Striga* germination assays and liquid chromatography–mass spectrometry analysis showed that root exudates of *ccd7* mutants were also SL deficient.

**Conclusions:**

Taken together, our results show the potential and feasibility of the use of the CRISPR/Cas9 system for targeted engineering of plant architecture and for elucidating the molecular underpinnings of architecture-related traits.

**Electronic supplementary material:**

The online version of this article (10.1186/s12870-018-1387-1) contains supplementary material, which is available to authorized users.

## Background

Technologies that facilitate efficient, robust, and precise engineering of the plant genome can be used for targeted improvement of crop traits, and will pave the way for increasing plant yield and improving food security [35]. Plant architecture is dynamically regulated by developmental and environmental factors, and has key effects on yield. For example, in the Green Revolution, random mutagenesis and harnessing of natural variants of key architecture genes in grain crops yielded varieties with shorter heights, resulting in significant improvement of crop productivity [40].

Genome engineering requires molecular scissors capable of making precise double strand breaks (DSBs) in the genome [41, 49]. Such DSBs are repaired by either the imprecise non-homologous end joining (NHEJ) repair or the precise homology directed repair (HDR) pathways [14, 45, 49]. Harnessing the cellular repair pathways of the DSBs, a variety of user-desired genetic outcomes can be generated. Different platforms of site-specific nucleases (SSNs) have been used to engineer the eukaryotic genomes including homing endonucleases, zinc finger nucleases (ZFNs), transcription activator-like effector nucleases (TALENs) [30]. A novel class of SSNs, clustered regularly interspaced palindromic repeats (CRISPR)/CRISPR associated (Cas) 9 system is profoundly revolutionizing our ability to engineer the plant genome [14, 18, 19, 26, 38, 41]. Recently, different CRISPR/Cas systems have been harnessed to edit and determine RNA levels, and for other RNA manipulations [5, 6, 34].

Strigolactones are a novel class of plant hormones that play an essential role in establishing plant architecture, determining the number of shoot branches/tillers and regulating the growth of primary and lateral roots [3, 21, 28, 48, 50]. SLs also participate in biotic and abiotic stress responses [17, 23, 46]. Furthermore, plant roots release SLs into the rhizosphere to trigger hyphal branching in mycorrhizal fungi for establishment of the beneficial arbuscular mycorrhizal symbiosis used by around 80% of land plants to improve nutrient uptake [9, 22]. However, seeds of root-parasitic weeds of the genus *Striga* perceive SLs as a germination signal ensuring the presence of a nearby host [53]. Infestation by *Striga hermonthica* and related parasitic plants causes enormous yield losses in many crops, such as cereals and different *Solanaceae* species, representing a severe problem for agriculture in sub-Saharan Africa, Southern Europe, the Middle East and Asia [37].

**Fig. 1:**
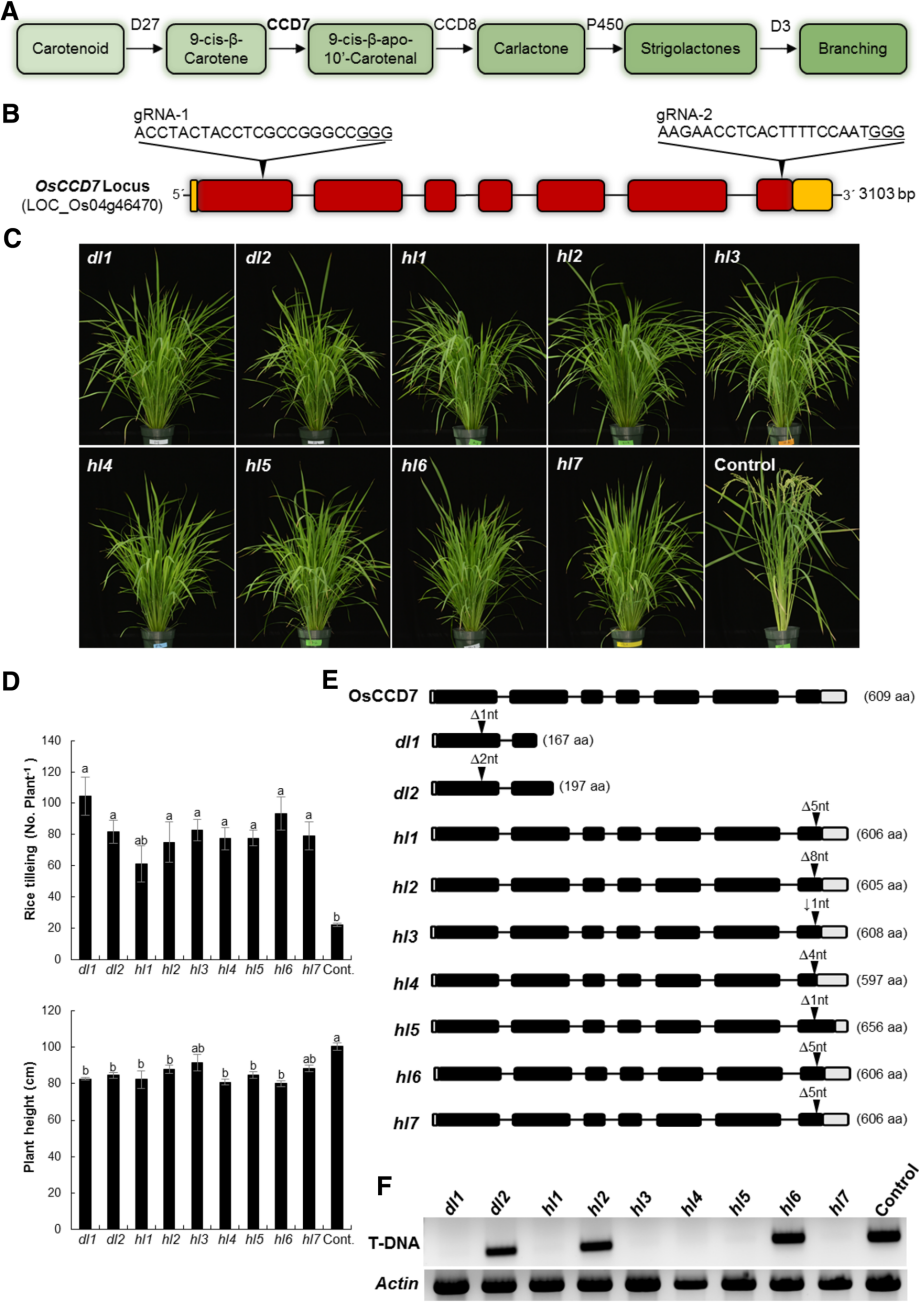
*OsCCD7* mutagenesis produced high-tillering and reduced height phenotypes: **a** Schematic of the strigolactone biosynthesis pathway showing branching/tillering inhibition activity. Carotenoid Cleavage Dioxygenase 7 (CCD7) catalyzes 9′-cis-β-carotene at the initial steps of SL biosynthesis. **b** Gene model of *OsCCD7*/ *D17* (Dwarf 17)/ *HTD1* (High-Tillering Dwarf1), also called MAX3 ortholog (LOC_Os04g46470). Two gRNA were used to target the *OsCCD7* locus. gRNA-1 was designed to target the 1st exon to produce a mutation similar to *d17*. The nomenclature used for these mutant lines is *dl (d17-like)*. Two T_0_ lines were produced, *dl1* and *dl2*. gRNA-2 was designed to target the 7th exon. The mutant lines produced were similar to *htd1* and these lines were named as *hl (htd1-like).* Seven independent lines were produced, *hl1* to *hl7*. The underlined GGG represents PAM sequence. **c** and **d** High-tillering and lower plant height phenotypes were observed for mutant plants. Tillering and plant height per plant were recorded from four plants per line (*n* = 4). All of the genotypes showed significant increases in tillers per plant and decreased plant height compared to control. **e** T_1_ generation of mutants were genotyped and mono-allelic lines were identified. Each of these mutations produced a protein variant. The nucleotide indels and protein alignments are shown in Additional file 1: Figure S2 and S3 respectively. **f** Analysis of the T_2_ generation showed some of the mutant lines do not carry a T-DNA. T-DNA-specific PCR analysis indicated that *dl1, hl1, hl3, hl4, hl5* and *hl7* are non-transgenic mutated plants. *Actin* PCR was done as a control

Analysis of SL-deficient and SL-perception mutants paved the way for the elucidation of the major steps in SL biosynthesis and signaling [3, 28, 51]. For example, the increased branching/tillering phenotype of *carotenoid cleavage dioxygenase 7* (*ccd7*) and *ccd8* mutants from *Arabidopsis thaliana*, pea (*Pisum sativum*), petunia (*Petunia hybrida*), and rice suggested the role of these enzymes in the biosynthesis of a shoot branching inhibitor that was later identified as SL [3, 39]. SLs are carotenoid-derivatives synthesized from all-*trans*-β-carotene via a pathway involving the all-*trans*/9-*cis*-β-carotene isomerase (DWARF27 in rice) that forms 9-*cis*-β-carotene (Fig. 1a) [4, 11]. In the next step, the stereospecific enzyme CCD7 cleaves 9-*cis*-β-carotene into the volatile β-ionone and 9-*cis*-β-apo-10′-carotenal [4, 12]. This *cis*-configured intermediate is the substrate of CCD8 that catalyzes a combination of reactions, including repeated deoxygenation and intramolecular rearrangements, which yield carlactone and a C_8_-product (ω-OH-(4-CH3) heptanal) (Fig. 1a) [4, 13]. Very recently, CCD8 enzymes have been also shown to produce 3-hydroxy-carlactone from accordingly hydroxylated, 9-*cis*-configured precursor [8]. Carlactone is the precursor of canonical and non-canonical SLs [28]. In Arabidopsis, carlactone is converted by a cytochrome P450 of the 711 clade (MAX1) into carlactonoic acid, followed by methylation by an unknown enzyme and hydroxylation by lateral branching oxidoreductase (LBO) into a yet unidentified SL [1, 10]. Rice MAX1 homologs convert carlactone into the known SLs 4-deoxyorobanchol and orobanchol, likely via carlactonoic acid [27, 57].

The first step in SL signal transduction is the binding of SL to the receptor, DWARF14 (D14) in rice, an α/β-hydrolase that hydrolyzes SL ligands and forms a covalent bond with one of the two hydrolysis products (D-Ring) [16, 24, 55]. These steps are accompanied by a conformational change that enables the interaction with an F-box protein (MAX2 in Arabidopsis; D3 in rice), which is a constituent of an SKP1-CUL1-F-box-protein (SCF)-type ubiquitin ligase complex, initiating the 26S proteasomal degradation of target transcription repressors [29, 44, 50, 58].

Targeted mutation of key enzymes in SL biosynthesis or perception can be used to engineer plant architecture in ways that improve yield. Moreover, in contrast to traditional, time-consuming approaches involving breeding natural alleles into elite varieties, genome editing provides a targeted, precise and rapid method to improve key plant traits in agronomically relevant, locally adapted varieties. OsCCD7 catalyzes a key step in SL biosynthesis, and an *OsCCD7* point mutation (C-T) in the Nanjing 6 background, *htd1 (high-tillering and dwarf 1*), causes a dwarf phenotype and production of a high number of tillers [56, 59]. This indicates the possibility of manipulating plant height and tillering, key agronomic traits, by genome editing of *OsCCD7*. In this work, we report the engineering of SL biosynthesis by CRISPR/Cas9-mediated mutation of *OsCCD7*. Our data provide a proof-of-principle for translational application of knowledge on SL biosynthesis for improvement of plant architecture.

## Methods

### Plant materials and vector construction

*Oryza sativa* L. ssp. *japonica* cv Nipponbare was used for all experiments. The pRGEB32 vector was used for callus transformations [14]. The expression of Cas9 was driven by *OsUbiquitin* promotor, and the gRNA was expressed as a polycistronic tRNA-gRNA under the *OsU3* promoter*.* The *OsCCD7* (*LOC_Os04g46470*) was used as a target locus. Two gRNAs were designed and transformed independently. gRNA-1 was designed to target the genomic sequence 289 to 308 bp (5′- ACCTACTACCTCGCCGGGCCGGG-3′); the underlined GGG represents the PAM sequence. gRNA-2 was designed to target the genomic sequence 2416 to 2435 bp (5′- AAGAACCTCACTTTTCCAATGGG-3′); the underlined GGG represents the PAM sequence. The potential off-targets were predicted using Cas-OFFinder [7] and no –off-targets were found for these sgRNAs. The void pRGEB32 vector lacking gRNA was transformed into Nipponbare and used as control. The high-tillering/dwarf *d3–1* and *d17–1* mutants, in Shiokari background (Ishikawa et al. 2005), were used for comparison.

### Rice transformation and mutant screening

*Agrobacterium*-mediated rice transformation was performed as described previously [14]. Transgenic rice plants were grown in a greenhouse at 28 °C. After 1 week when plants were established on soil, DNA was extracted from leaf samples. PCR was done using gene-specific primers. Purified PCR products were cloned using the CloneJET PCR Cloning Kit (K1231). Sanger sequencing was done for at least 10 colonies, to analyze the mutation.

### Rice tillering bioassays

Rice seeds were surface-sterilized with 2.5% sodium hypochlorite for 10 min. The seeds were then washed thoroughly with sterile MilliQ water and imbibed in water for 2 days at 30 °C in the dark. The pre-germinated seeds were shifted to 90-mm Petri dishes on a filter paper moistened with 5 ml ½-strength Murashige and Skoog medium [36]. The sealed plates were kept at 30 °C for one more night to develop small seedlings. The plates with small seedlings were kept under fluorescent white light (130–180 μM m^− 2^ s^− 1^) for 7 days. One-week-old uniform seedlings were selected to grow in a microfuge tube, fixed on top of 50-ml tubes (one seedling per tube, with five replications) filled with half-strength modified Hoagland nutrient solution [25]. After 1 week, the rice plants were supplemented with GR24 at 2.5 μM. The GR24 was added six times, twice a week. Number of tillers per plant and plant height of all lines including control, *d3*, and *d17* were measured at final harvest.

### Quantitation of SLs from rice root exudates

One week old rice seedlings were established as described above and grown hydroponically in a growth cabinet with half-strength modified Hoagland nutrient solution under normal phosphorus (Pi) supply for 1 week. Then, rice seedlings were kept in a Pi-deficient nutrient solution for another week. On the day of root exudate collection, rice seedlings were first refreshed with Pi-deficient Hoagland nutrient solution for 6 h, and root exudates were then collected from each tube. SLs were then extracted from root exudates for LC/MS-MS analysis and *Striga* bioassays. For this purpose, SPE C_18_ columns (Grace Pure) was pre-conditioned by solvation (6 ml of methanol) and equilibration (6 ml of water). After adding the internal standard D_6_–5-Deoxystrigol (0.672 ng per 50 ml root exudate), root exudates were run through the preconditioned SPE C_18_ column. After washing with 6 ml of water, SLs were eluted with 5 ml of acetone. The SL fraction (acetone-water solution) was concentrated to SL aqueous solution (∼1 ml), followed by extraction with 1 ml of ethyl acetate. Then 750 μl of SL-enriched organic phase was transferred to a 1.5-ml tube and evaporated to dryness. The sample was re-dissolved in 100 μl of acetonitrile:water (25:75, v:v) and filtered through a 0.22-μm filter for LC-MS/MS analysis. SLs were analyzed using HPLC-Q-Trap-MS/MS with MRM mode. Chromatographic separation was achieved on an Acquity UPLC BEH C_18_ column (50 × 2.1 mm; 1.7 μm; Waters). Mobile phases consisted of water:acetonitrile (95:5, v:v) and acetonitrile, both containing 0.1% formic acid.

### *Striga hermonthica* bioassays

The *Striga hermonthica* seed germination bioassay was conducted as described previously (Jamil et al., 2012). The *Striga* seeds were preconditioned for 10 days at 30 °C under moist conditions. The pre-conditioned *Striga* seeds were supplied with 50 μl acetone-free SL extract collected from the root exudates for each mutant line as described above. Wild type and the *d17* mutant were included as positive and negative controls, respectively. After SL application, *Striga* seeds were incubated at 30 °C in the dark for 2 days. Germinated (seeds with radicle) and non-germinated seeds were counted under a binocular microscope to calculate germination rate (%).

## Results

### Targeted engineering of *CCD7* mutants in rice via CRISPR/Cas9

We used CRISPR/Cas9 for targeted mutagenesis of *OsCCD7* to generate variants for translational research and to enhance our understanding of the diverse functions of this protein. The *OsCCD7* gene (*LOC_Os04g46470*) has 7 exons encoding a protein of 609 amino acids (Fig. 1b), which mediates a key step in SL biosynthesis (Fig. 1a). For targeted mutagenesis of *OsCCD7*, we engineered two sgRNAs, sgRNA-1 targeting the 1^st^ exon and sgRNA-2 targeting 7^th^ exon (Fig. 1b).

After regeneration of T_0_ plants from the calli, we examined the resulting plants for mutations in *OsCCD7*. We recovered 22 T_0_ transgenic plants corresponding to sgRNA-1 and 17 T_0_ transgenic plants corresponding to sgRNA-2 and genotyped these plants by PCR amplifying the region encompassing the target site of the sgRNAs. PCR amplicons were cloned and sequenced. For sgRNA-1, 8/22 plants showed bi-allelic mutations of the target site with formation of insertion/deletion mutations (indels) including deletions of 1–27 bp. Similarly, for sgRNA-2, all of the plants were bi-allelic with indels. Some plants, however, exhibited only monoallelic mutations. These data show the high efficiency of targeted mutagenesis for *CCD7* by gRNAs targeting the 1^st^ and 7^th^ exons. We used the nomenclature *d17-like* (*dl*) for mutants produced by sgRNA-1. Two mono-allelic mutants, *dl1* and *dl2* with one- and two-bp deletions, respectively, were used for further studies (Additional file 1: Figure S1A). For sgRNA-2 mutants, we used the nomenclature *htd1-like* (*hl*). Seven bi-allelic mutants *hl1*–*hl7* were used for further studies (Additional file 1: Figure S1A).

### The *ccd7* mutants exhibit increased tillering and reduced height phenotypes

Our genotyping data revealed the presence of several *ccd7* mutants resulting in complete or partial functional knockout phenotypes. Phenotyping the T_0_ plants can accelerate functional analysis, but the presence of two alleles can complicate interpretation. To examine this, we phenotyped the T_0_ plants for the number of tillers and plant height (Additional file 1: Figure S1B and C). Our data indicate that the mutants produced increased numbers of tillers and reduced plant height, reminiscent of the *ccd7* mutants isolated by conventional methods in other plant species (Additional file 1: Figure S1B and C). *hl2* and *hl5* showed the highest numbers of tillers per plant, while *dl2* and *hl1* exhibited the maximum reduction in plant height among all mutants (Additional file 1: Figure S1C). However, *hl4* showed high tillering accompanied with less reduced height, (Additional file 1: Figure S1C).

Most of the *hl* mutations observed were bi-allelic in the T_0_ generation. To analyze and correlate the phenotypes with a particular protein variant, T_1_ plants were genotyped by sequencing. Mono-allelic, homozygous mutant plants were identified and used for further studies. All of the mutant lines, including *hl4*, showed pronounced high tillering and reduced plant height (Fig. 1c, d). Among these mutant lines, *dl1* and *hl6* showed the highest number of tillers per plant (Fig. 1d). Each of the mutant lines harbors a particular mutation that leads to a CCD7 protein variant (Fig. 1e, Additional file 1: Figure S2). These protein variants produce variation in tillering and plant height (Fig. 1d).

One advantage of CRISPR/Cas9 mutagenesis is that the mutation can be segregated away from the T-DNA expression construct used to produce Cas9 and the gRNA. To analyze whether these lines harbor a T-DNA, we examined the T_2_ generation of these mutants. We found some of the plants have no T-DNA but do have mutations in *CCD7* (Fig. 1f). This further showed that non-transgenic mutated plants of agricultural importance can be produced.

### *ccd7* mutants exhibit impaired SL biosynthesis

**Fig. 2:**
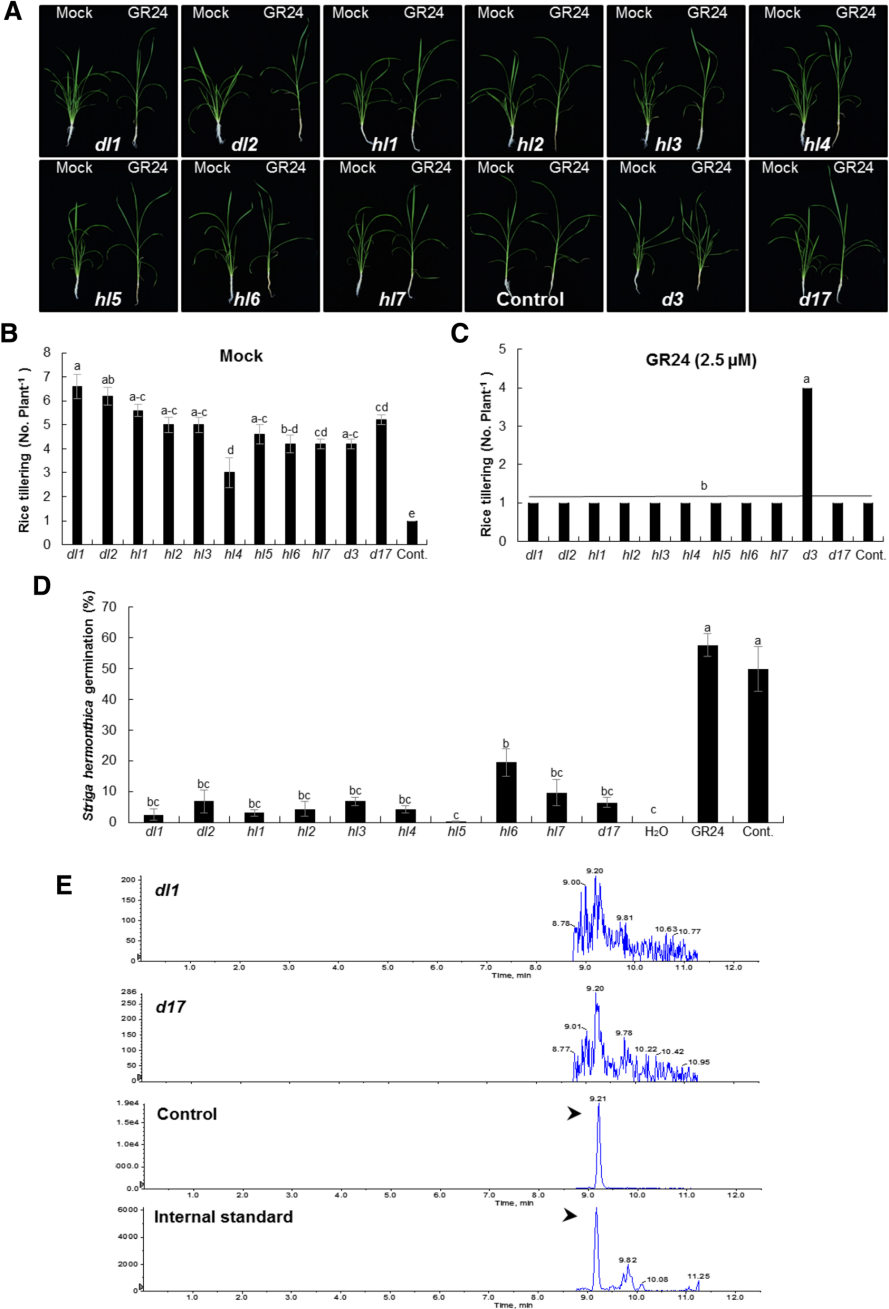
The *ccd7* mutants showed impaired SL biosynthesis and decreased SL-dependent biological activity: **a** GR24 treatment rescued the tillering phenotype of all T_1_-mutant lines. One-week-old seedlings were grown in 50-ml tubes and GR24, a synthetic SL analog, was applied twice a week at 2.5 μM for 3 weeks. Cas9ox (Cont.), the SL-deficient mutant *d17*, and the SL-perception mutant *d3* were used as controls. Tillering inhibition by GR24 feeding indicated that the high-tillering phenotype appeared because of lack of SLs biosynthesis. **b-c** Tillers per plant were recorded with or without GR24 treatment after 3 weeks of application. No. of tillers per plant were significantly reduced after GR24 treatment. Bars represent means ±SE (*n* = 6). Means not sharing a letter in common differ significantly at *P*_0.05_. **d** *Striga* germination bioassay was conducted to measure SL bioactivity in T_1_ mutant lines. The rice plants were grown under normal conditions for 3 weeks. Then in 4th week each line was grown under phosphate-deficient conditions for another week. The SLs were extracted from root exudates of each line and applied to pre-conditioned *Striga* seeds. Very low *Striga* seed germination was observed in mutants compared to control, indicating SL deficiency in these lines. Graph shows percent *Striga* germination in response to root exudates collected from each line. Cas9ox (Cont.) and *d17* were used as control plant lines; in addition, the SL analog GR24 and H_2_O were applied to *Striga* seeds with no root extracts for controls to show maximum and minimum germination. **e** LC–MS/MS analysis using multiple reaction monitoring (MRM) of rice root exudates. The MRM transitions for various SLs from root exudates of control, *d17* and *ccd7* T_1_-mutant lines were observed. All of the mutants and *d17* did not show any detectable signals for SLs. The chromatogram showed peaks for 4-deoxystrigol in the internal standard and control. One of the mutants (*dl1*) chromatogram is shown as an example

To further show that the *ccd7* mutant phenotype results from defects in SL biosynthesis, we tested whether treatment of the *ccd7* mutants with the synthetic SL analogue GR24 could restore their phenotypes to wild type. Progeny seeds were germinated on filter paper in Petri dishes and one-week-old seedlings were grown in 50-ml tubes for another week. The synthetic SL analogue GR24 was applied at 2.5 μM concentration for 3 weeks, then tiller numbers were counted. Application of GR24 to the *ccd7* mutants decreased tiller numbers to wild-type levels (Fig. 2a–c). The observed number of tillers of *ccd7* mutants was 7 tillers per plant on average in untreated (Mock) plants and this decreased to 1 tiller per plant with GR24 treatment (Fig. 2a–c).

To confirm the SL deficiency of the *ccd7* mutants, we quantified the levels of SLs in the corresponding root exudates, using liquid chromatography quadruple time-of-flight tandem mass spectrometry (LC-MS/MS). The LC-MS/MS data indicated that all the mutants showed defective and very poor synthesis of strigolactone, which cannot be detected and quantified by LC-MS/MS (Fig. 2e).

### The *ccd7* mutants affected *Striga* germination

SLs induce the germination of parasitic seeds of the *Orobancheaceae* family, including *Striga*; therefore, we measured the ability of exudates from *ccd7* mutant roots to stimulate the germination of *Striga* seeds. We found a significant reduction in *Striga* germination with the root exudates of *ccd7* mutants as compared to wild type (Fig. 2d). Although the *ccd7* mutants exhibited a defective and extremely poor levels of SL production as compared with controls but they induced some *Striga* seed germination. However, this *Striga* seed germination by the root exudates of *ccd7* mutants was significantly less than that of control and the standard SL analog GR24. The low *Striga* seed germination by *ccd7* mutants might be due to its highly sensitive perception protein especially ShHTL7 that can detect SL in traces which could not be detectable by LC-MS/MS.

## Discussion

Use of the CRISPR/Cas9 system to precisely manipulate the plant genome in a user-defined manner opens myriad possibilities for translational applications in agriculture [5, 6, 19, 20, 34, 42, 49]. Genes controlling plant architecture are prime targets for engineering plant varieties with higher yields; however, to avoid unintended effects, such engineering requires a strong understanding of the mechanisms by which such genes function. In recent years, multiple lines of inquiry have provided a substantial understanding on the biology of SLs, including their biosynthesis, transport, signaling, and interactions with other hormonal responses [43, 51]. SLs control shoot branching and stimulate the germination of parasitic plants; therefore, the manipulation of SL biosynthesis may reduce crop losses and increase yield [15, 33]. For example, reduction of SLs in plants and root exudates could compromise the germination of parasitic plant seeds, and thus reduce crop losses. Indeed, reduction of SL production has shown promise in plant species including rice, pea, fava bean (*Vicia faba*), tomato (*Solanum lycopersicum*), and maize (*Zea mays*) [28, 47]. However, significant reductions in SL levels may compromise other factors, such as the formation of arbuscular mycorrhizal symbioses, and thereby ultimately prove to be a counter-productive strategy [28, 47]. Moreover, finding germplasm of a given crop species with reduced and fine-tuned SL production remains challenging due to the limited genetic diversity of crop varieties. The CRISPR/Cas9 system can be used to produce the much-needed genetic diversity and generate varieties with fine-tuned levels of proteins or metabolites such as SLs.

Here, we employed the CRISPR/Cas9 system for targeted engineering of *CCD7* to produce rice mutants with reduced SL biosynthesis. For this purpose, we designed two sgRNAs that target two exons in *CCD7* with the aim of generating knockout phenotypes. The deletion of one or two nucleotides at the sgRNA-1 target site in the first exon produced the alleles *dl1* and *dl2*, respectively, and resulted in truncated CCD7 proteins. Targeting the terminal region, the last 7^th^ exon by sgRNA-2 produced seven different alleles labelled *hl1 to hl7*. The C-terminus of CCD7 protein is an important region to target as any substitution in this region might disrupt the function of the protein [59]. CCD7 contains four highly conserved histidine residues, including H603 that is located in the C-terminus. These four residues are responsible for coordinating the catalytic iron in the reaction center and, hence, are essential for the enzymatic activity of CCDs [2, 31, 52]. Any mutation upstream of this histidine could affect the function of the protein. Both type of alleles, i.e. *dl* and *hl* gave rise to a significant reduction in plant height and an increase in tiller number (Fig. 1c and d). The *dl* and *hl* high-tillering phenotype was similar to that reported for *htd-1* and *dit1* resulting from deletions of one and two nucleotides in the first exon and a C to A mutation generating a stop codon in sixth exon, respectively [32, 59]. However, targeting the 2^nd^ exon in rice *CCD7* by CRISPR/Cas9 has been recently shown to cause a more pronounced high-tillering phenotype with an average of 145 tillers per plant [54].

In addition, the plant height of *hl1 to hl7* lines generated here was reduced by only 10–20%, which is less pronounced compared with the dwarfism previously reported for rice *ccd7* mutants [32, 54, 59]. These differences might be due to different cultivar backgrounds. However, we have also observed a variation in the number of tillers per plant among different mutants, with *dl1* producing the highest number of tillers (104 tillers per plant) and *hl1* the lowest (61 tillers per plant). It can be assumed that these difference are a result of regeneration from tissue culture. In the next segregating generation, some of mutants were lacking the T-DNA (Fig. 1f), which confirms the production of non-transgenic mutant plants with altered plant architecture.

To confirm that the phenotypes observed were caused by SL deficiency, we used the synthetic SL analogue GR24 to restore WT tillering phenotype in *dl* and *hl* mutants. With the application of SL analog, all the high tillering mutants showed reduced tillering equal to wild type control, indicating it as SL dependent phenotype. Next, we have quantified SL levels in root exudates by LC-MS using D_6_–5-Deoxystrigol as internal standard. SLs were not detected in the root exudates of any of these mutants, again showing the interruption of SLs biosynthesis. This fact was further confirmed by *Striga hermonthica* seed germination assay, a parasitic plant highly sensitive to picomolar concentrations of SLs. Root exudates of the *dl* and *hl* mutants showed very low *Striga* seed germinating activity compared to that of wild type or to the SL analog GR24 control. Collectively, the consistency of these results indicated that the observed high tillering phenotype was due to the disruption of *CCD7*. The determination of other factors or machineries that might be at paly requires further studies.

## Conclusions

The findings of the present study provide a compelling proof-of-concept on the utility of the CRISPR/Cas9 system in translational research for targeted improvement of plant architecture traits. This study shows that targeted engineering of *CCD7*, and possibly other SLs biosynthesis genes, and fine-tuning of SL levels would produce altered plant architecture in diverse crop species to improve yield and resilience. In this example, developing rice with reduced levels of *CCD7* may help to fine-tune the levels of SLs which lead to altered plant architecture especially tillering to improve crop yield and might lower the risk of *Striga* infection.

## Additional file

Additional file 1: Supplementary Information. (DOCX 65982 kb)

## Abbreviations

Cas9: CRISPR-associated protein 9
CCD7: *CAROTENOID CLEAVAGE DIOXYGENASE 7*
*CRISPR*: Clustered Regularly Interspaced Short Palindromic Repeats
*D17*: (Dwarf 17)
*DL*: *D17-Like*
gRNA: Guide RNA
*HL*: *HTD1-Like*
*HTD1*: High-Tillering Dwarf1
SL: Strigolactone

## References

[1] Abe S, Sado A, Tanaka K, Kisugi T, Asami K, Ota S, Kim HI, Yoneyama K, Xie X, Ohnishi T, Seto Y, Yamaguchi S, Akiyama K, Yoneyama K, Nomura T (2014). Carlactone is converted to carlactonoic acid by MAX1 in Arabidopsis and its methyl ester can directly interact with AtD14 in vitro. Proc Natl Acad Sci U S A.

[2] Ahrazem O, Gomez-Gomez L, Rodrigo MJ, Avalos J, Limon MC (2016). Carotenoid cleavage Oxygenases from microbes and photosynthetic organisms: features and functions. Int J Mol Sci.

[3] Al-Babili S, Bouwmeester HJ (2015). Strigolactones, a novel carotenoid-derived plant hormone. Annu Rev Plant Biol.

[4] Alder A, Jamil M, Marzorati M, Bruno M, Vermathen M, Bigler P, Ghisla S, Bouwmeester H, Beyer P, Al-Babili S (2012). The path from beta-carotene to carlactone, a strigolactone-like plant hormone. Science.

[5] Ali Z, Mahas A, Mahfouz M (2018). CRISPR/Cas13 as a tool for RNA interference. Trends Plant Sci.

[6] Aman R, Ali Z, Butt H, Mahas A, Aljedaani F, Khan MZ, Ding S, Mahfouz M (2018). RNA virus interference via CRISPR/Cas13a system in plants. Genome Biol.

[7] Bae S, Park J, Kim JS (2014). Cas-OFFinder: a fast and versatile algorithm that searches for potential off-target sites of Cas9 RNA-guided endonucleases. Bioinformatics.

[8] Baz L, Mori N, Mi J, Jamil M, Kountche BA, Guo X, Balakrishna A, Jia KP, Vermathen M, Akiyama K, Al-Babili S. 3-Hydroxycarlactone, a novel product of the Strigolactone biosynthesis Core pathway. Mol Plant. 20182052(18):30215-6. 10.1016/j.molp.2018.06.008. 10.1016/j.molp.2018.06.008 29969682

[9] Bonfante P, Genre A (2015). Arbuscular mycorrhizal dialogues: do you speak ‘plantish’ or ‘fungish’?. Trends Plant Sci.

[10] Brewer PB, Yoneyama K, Filardo F, Meyers E, Scaffidi A, Frickey T, Akiyama K, Seto Y, Dun EA, Cremer JE, Kerr SC, Waters MT, Flematti GR, Mason MG, Weiller G, Yamaguchi S, Nomura T, Smith SM, Yoneyama K, Beveridge CA (2016). LATERAL BRANCHING OXIDOREDUCTASE acts in the final stages of strigolactone biosynthesis in Arabidopsis. Proc Natl Acad Sci U S A.

[11] Bruno M, Al-Babili S (2016). On the substrate specificity of the rice strigolactone biosynthesis enzyme DWARF27. Planta.

[12] Bruno M, Hofmann M, Vermathen M, Alder A, Beyer P, Al-Babili S (2014). On the substrate- and stereospecificity of the plant carotenoid cleavage dioxygenase 7. FEBS Lett.

[13] Bruno M, Vermathen M, Alder A, Wust F, Schaub P, van der Steen R, Beyer P, Ghisla S, Al-Babili S (2017). Insights into the formation of carlactone from in-depth analysis of the CCD8-catalyzed reactions. FEBS Lett.

[14] Butt H, Eid A, Ali Z, Atia MAM, Mokhtar MM, Hassan N, Lee CM, Bao G, Mahfouz MM (2017). Efficient CRISPR/Cas9-mediated genome editing using a chimeric single-guide RNA molecule. Front Plant Sci.

[15] Conn CE, Bythell-Douglas R, Neumann D, Yoshida S, Whittington B, Westwood JH, Shirasu K, Bond CS, Dyer KA, Nelson DC (2015). PLANT EVOLUTION. Convergent evolution of strigolactone perception enabled host detection in parasitic plants. Science.

[16] de Saint GA, Clave G, Badet-Denisot MA, Pillot JP, Cornu D, Le Caer JP, Burger M, Pelissier F, Retailleau P, Turnbull C, Bonhomme S, Chory J, Rameau C, Boyer FD (2016). An histidine covalent receptor and butenolide complex mediates strigolactone perception. Nat Chem Biol.

[17] Decker EL, Alder A, Hunn S, Ferguson J, Lehtonen MT, Scheler B, Kerres KL, Wiedemann G, Safavi-Rizi V, Nordzieke S, Balakrishna A, Baz L, Avalos J, Valkonen JPT, Reski R, Al-Babili S (2017). Strigolactone biosynthesis is evolutionarily conserved, regulated by phosphate starvation and contributes to resistance against phytopathogenic fungi in a moss, Physcomitrella patens. New Phytol.

[18] Doudna JA, Charpentier E (2014). Genome editing. The new frontier of genome engineering with CRISPR-Cas9. Science.

[19] Eid A, Alshareef S, Mahfouz MM (2018). CRISPR base editors: genome editing without double-stranded breaks. Biochem J.

[20] Eid A, Mahfouz MM (2016). Genome editing: the road of CRISPR/Cas9 from bench to clinic. Exp Mol Med.

[21] Gomez-Roldan V, Fermas S, Brewer PB, Puech-Pages V, Dun EA, Pillot JP, Letisse F, Matusova R, Danoun S, Portais JC, Bouwmeester H, Becard G, Beveridge CA, Rameau C, Rochange SF (2008). Strigolactone inhibition of shoot branching. Nature.

[22] Gutjahr C, Paszkowski U (2013). Multiple control levels of root system remodeling in arbuscular mycorrhizal symbiosis. Front Plant Sci.

[23] Ha CV, Leyva-Gonzalez MA, Osakabe Y, Tran UT, Nishiyama R, Watanabe Y, Tanaka M, Seki M, Yamaguchi S, Dong NV, Yamaguchi-Shinozaki K, Shinozaki K, Herrera-Estrella L, Tran LS (2014). Positive regulatory role of strigolactone in plant responses to drought and salt stress. Proc Natl Acad Sci U S A.

[24] Hamiaux C, Drummond RS, Janssen BJ, Ledger SE, Cooney JM, Newcomb RD, Snowden KC (2012). DAD2 is an alpha/beta hydrolase likely to be involved in the perception of the plant branching hormone, strigolactone. Curr Biol.

[25] Hoagland DR, Arnon DI. The water culture method for growing plants without soil. Agricultural Experiment Station, University of California, Berkeley. Circular. 1950;347:1–32.

[26] Hsu PD, Lander ES, Zhang F (2014). Development and applications of CRISPR-Cas9 for genome engineering. Cell.

[27] Iseki M, Shida K, Kuwabara K, Wakabayashi T, Mizutani M, Takikawa H, Sugimoto Y (2018). Evidence for species-dependent biosynthetic pathways for converting carlactone to strigolactones in plants. J Exp Bot.

[28] Jia KP, Baz L, Al-Babili S (2018). From carotenoids to strigolactones. J Exp Bot.

[29] Jiang L, Liu X, Xiong G, Liu H, Chen F, Wang L, Meng X, Liu G, Yu H, Yuan Y, Yi W, Zhao L, Ma H, He Y, Wu Z, Melcher K, Qian Q, Xu HE, Wang Y, Li J (2013). DWARF 53 acts as a repressor of strigolactone signalling in rice. Nature.

[30] Kim H, Kim JS (2014). A guide to genome engineering with programmable nucleases. Nat Rev Genet.

[31] Kloer DP, Ruch S, Al-Babili S, Beyer P, Schulz GE (2005). The structure of a retinal-forming carotenoid oxygenase. Science.

[32] Kulkarni KP, Vishwakarma C, Sahoo SP, Lima JM, Nath M, Dokku P, Gacche RN, Mohapatra T, Robin S, Sarla N, Seshashayee M, Singh AK, Singh K, Singh NK, Sharma RP (2014). A substitution mutation in OsCCD7 cosegregates with dwarf and increased tillering phenotype in rice. J Genet.

[33] Lopez-Raez JA, Matusova R, Cardoso C, Jamil M, Charnikhova T, Kohlen W, Ruyter-Spira C, Verstappen F, Bouwmeester H (2009). Strigolactones: ecological significance and use as a target for parasitic plant control. Pest Manag Sci.

[34] Mahas A, Neal Stewart C, Mahfouz MM (2018). Harnessing CRISPR/Cas systems for programmable transcriptional and post-transcriptional regulation. Biotechnol Adv.

[35] Moshelion M, Altman A (2015). Current challenges and future perspectives of plant and agricultural biotechnology. Trends Biotechnol.

[36] Murashige T, Skoog F (1962). A revised medium for rapid growth and bioassays with tobacco tissue cultures. Physiol Plant.

[37] Parker C (2009). Observations on the current status of Orobanche and Striga problems worldwide. Pest Manag Sci.

[38] Ran FA, Hsu PD, Lin CY, Gootenberg JS, Konermann S, Trevino AE, Scott DA, Inoue A, Matoba S, Zhang Y, Zhang F (2013). Double nicking by RNA-guided CRISPR Cas9 for enhanced genome editing specificity. Cell.

[39] Ruyter-Spira C, Al-Babili S, van der Krol S, Bouwmeester H (2013). The biology of strigolactones. Trends Plant Sci.

[40] Sakamoto T, Matsuoka M (2004). Generating high-yielding varieties by genetic manipulation of plant architecture. Curr Opin Biotechnol.

[41] Sander JD, Joung JK (2014). CRISPR-Cas systems for editing, regulating and targeting genomes. Nat Biotechnol.

[42] Schiml S, Puchta H (2016). Revolutionizing plant biology: multiple ways of genome engineering by CRISPR/Cas. Plant Methods.

[43] Seto Y, Yamaguchi S (2014). Strigolactone biosynthesis and perception. Curr Opin Plant Biol.

[44] Soundappan I, Bennett T, Morffy N, Liang Y, Stanga JP, Abbas A, Leyser O, Nelson DC (2015). SMAX1-LIKE/D53 family members enable distinct MAX2-dependent responses to Strigolactones and Karrikins in Arabidopsis. Plant Cell.

[45] Symington LS, Gautier J (2011). Double-strand break end resection and repair pathway choice. Annu Rev Genet.

[46] Torres-Vera R, Garcia JM, Pozo MJ, Lopez-Raez JA (2014). Do strigolactones contribute to plant defence?. Mol Plant Pathol.

[47] Tsuchiya Y, Yoshimura M, Hagihara S (2018). The dynamics of strigolactone perception in Striga hermonthica: a working hypothesis. J Exp Bot.

[48] Umehara M, Hanada A, Yoshida S, Akiyama K, Arite T, Takeda-Kamiya N, Magome H, Kamiya Y, Shirasu K, Yoneyama K, Kyozuka J, Yamaguchi S (2008). Inhibition of shoot branching by new terpenoid plant hormones. Nature.

[49] Voytas DF, Gao C (2014). Precision genome engineering and agriculture: opportunities and regulatory challenges. PLoS Biol.

[50] Waldie T, McCulloch H, Leyser O (2014). Strigolactones and the control of plant development: lessons from shoot branching. Plant J.

[51] Waters MT, Gutjahr C, Bennett T, Nelson DC (2017). Strigolactone signaling and evolution. Annu Rev Plant Biol.

[52] White MD, Flashman E (2016). Catalytic strategies of the non-heme iron dependent oxygenases and their roles in plant biology. Curr Opin Chem Biol.

[53] Xie X, Yoneyama K, Yoneyama K (2010). The strigolactone story. Annu Rev Phytopathol.

[54] Yang X, Chen L, He J, Yu W (2017). Knocking out of carotenoid catabolic genes in rice fails to boost carotenoid accumulation, but reveals a mutation in strigolactone biosynthesis. Plant Cell Rep.

[55] Yao R, Ming Z, Yan L, Li S, Wang F, Ma S, Yu C, Yang M, Chen L, Chen L, Li Y, Yan C, Miao D, Sun Z, Yan J, Sun Y, Wang L, Chu J, Fan S, He W, Deng H, Nan F, Li J, Rao Z, Lou Z, Xie D (2016). DWARF14 is a non-canonical hormone receptor for strigolactone. Nature.

[56] Zhang B, Tian F, Tan L, Xie D, Sun C (2011). Characterization of a novel high-tillering dwarf 3 mutant in rice. J Genet Genomics.

[57] Zhang Y, van Dijk AD, Scaffidi A, Flematti GR, Hofmann M, Charnikhova T, Verstappen F, Hepworth J, van der Krol S, Leyser O, Smith SM, Zwanenburg B, Al-Babili S, Ruyter-Spira C, Bouwmeester HJ (2014). Rice cytochrome P450 MAX1 homologs catalyze distinct steps in strigolactone biosynthesis. Nat Chem Biol.

[58] Zhou F, Lin Q, Zhu L, Ren Y, Zhou K, Shabek N, Wu F, Mao H, Dong W, Gan L, Ma W, Gao H, Chen J, Yang C, Wang D, Tan J, Zhang X, Guo X, Wang J, Jiang L, Liu X, Chen W, Chu J, Yan C, Ueno K, Ito S, Asami T, Cheng Z, Wang J, Lei C, Zhai H, Wu C, Wang H, Zheng N, Wan J (2013). D14-SCF (D3)-dependent degradation of D53 regulates strigolactone signalling. Nature.

[59] Zou J, Zhang S, Zhang W, Li G, Chen Z, Zhai W, Zhao X, Pan X, Xie Q, Zhu L (2006). The rice HIGH-TILLERING DWARF1 encoding an ortholog of Arabidopsis MAX3 is required for negative regulation of the outgrowth of axillary buds. Plant J.

