# Supplementary material for: Engineering plant architecture via CRISPR/Cas9-mediated alteration of strigolactone biosynthesis
Source: BMC Plant Biol. 2018 Aug 29;18:174. doi: 10.1186/s12870-018-1387-1 (PMC6116466; doi:10.1186/s12870-018-1387-1)
Supplement: Supplementary file 1 — Supplementary Information. (DOCX 65982 kb) [file 12870_2018_1387_MOESM1_ESM.docx]

**Supplementary Information**

**Engineering Plant Architecture via CRISPR/Cas9-mediated Alteration of Strigolactone Biosynthesis**

Haroon Butt ^1,3^, Muhammad Jamil ^2,3^, Jian You Wang^2^, Salim Al-Babili^2,*^ and Magdy Mahfouz^1,*^

^1^ Laboratory for Genome Engineering, Division of Biological Sciences, 4700 King Abdullah University of Science and Technology, Thuwal 23955-6900, Saudi Arabia

^2^ King Abdullah University of Science and Technology (KAUST), Biological and Environmental Sciences and Engineering Division,The Bioactives Lab, Thuwal 23955-6900, Saudi Arabia

^*^ Correspondence to:

[Salim.babili@kaust.edu.sa](mailto:Salim.babili@kaust.edu.sa) and [magdy.mahfouz@kaust.edu.sa](mailto:magdy.mahfouz@kaust.edu.sa)

^3^ These authors contributed equally to this work

**Key words:** genome editing, CRISPR/Cas9, strigolactones, plant architecture, Carotenoids, Carotenoid cleavage dioxygenases, CCD7

A

***dl1***

***dl2***

***hl1***

***hl2***

***hl3***

***hl4***

***hl5***

***hl6***

***hl7***

5’-CGGCCGGCACCTACTACCTCGCCGG-CCGGGCATCT-3’

5’-CGGCCGGCACCTACTACCTCGCCG--CCGGGCATCT-3’

5’-AGGTGCCAAAGAACCTCACTTTTC-AATGGGATTCC-3’

5’-AGGTGCCAAAGAACCTCACT-----AATGGGATTCC-3’

5’-AGGTGCCAAAGAACCTCACTTTTC-AATGGGATTCC-3’

5’-AGGTGCCAAAGAACCTCACT----CAATGGGATTCC-3’

5’-AGGTGCCAAAGAACCTCA--------ATGGGATTCC-3’

5’-AGGTGCCAAAGAACCTCACTTTA---AA-TGGGATTCC-3’

5’-AGGTGCCAAAGAACCTCACTTT-TCCAAATGGGATTCC-3’

5’-AGGTGCCAAAGAACCTCACTTT--CAATGGGATTCC-3’

5’-AGGTGCCAAAGAACCTCACTT----AATGGGATTCC-3’

5’-AGGTGCCAAAGAACCTCACTTTTC-AATGGGATTCC-3’

5’-AGGTGCCAAAGAACCTCACTTTT--AATGGGATTCC-3’

5’-AGGTGCCAAAGAACCTCACTTTTCAAATGGGATTCC-3’

5’-AGGTGCCAAAGAACCTCACTTTT-----GGGATTCC-3’

5’-AGGTGCCAAAGAACCTCAC-----CAATGGGATTCC-3’

5’-AGGTGCCAAAGAACCT------------GGGATTCC-3’

**5’-CGGCCGGCACCTACTACCTCGCCGGGCCGGGCATCT.......//......AGGTGCCAAAGAACCTCACTTTTCCAATGGGATTCC-3’**

**WT**

B


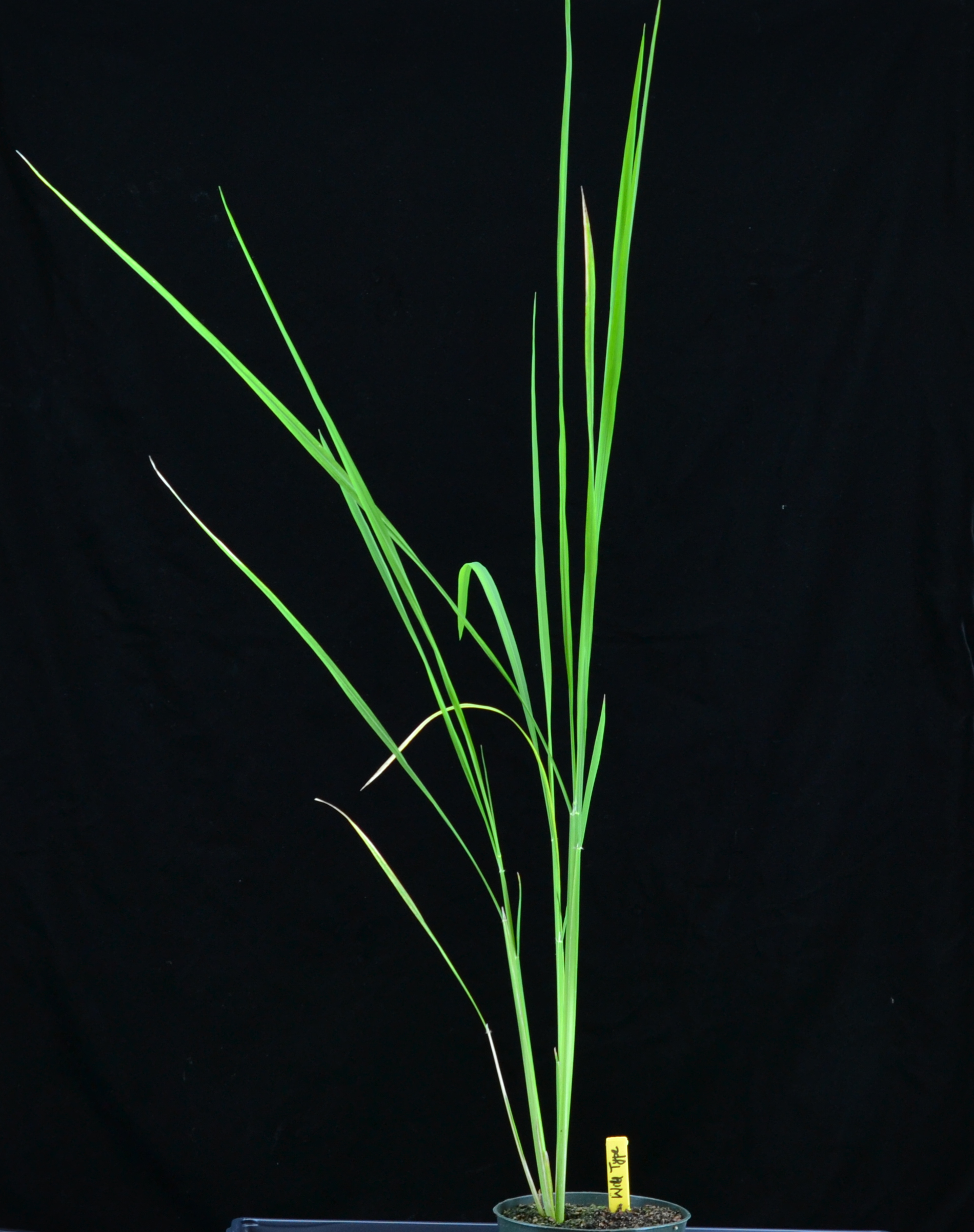

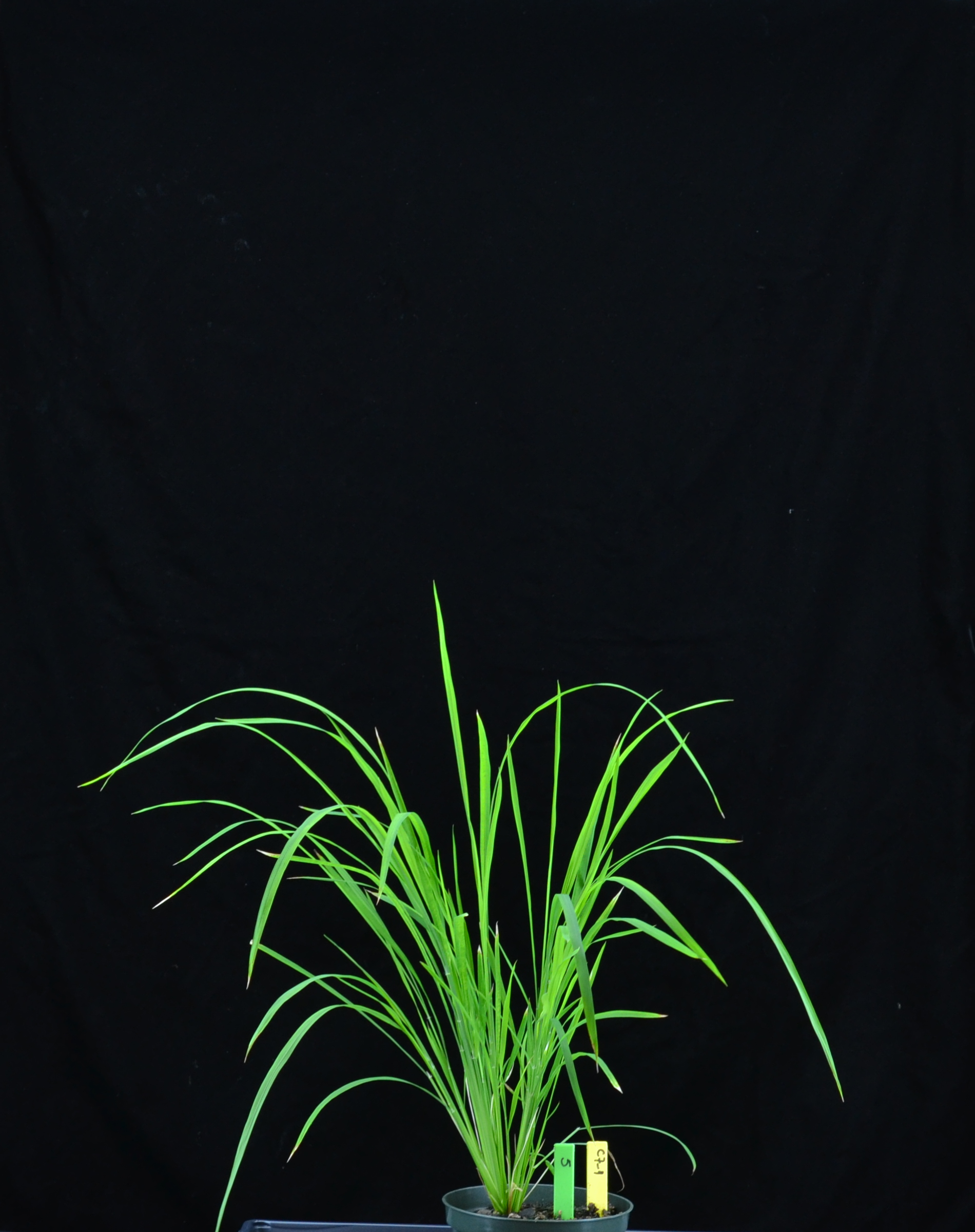

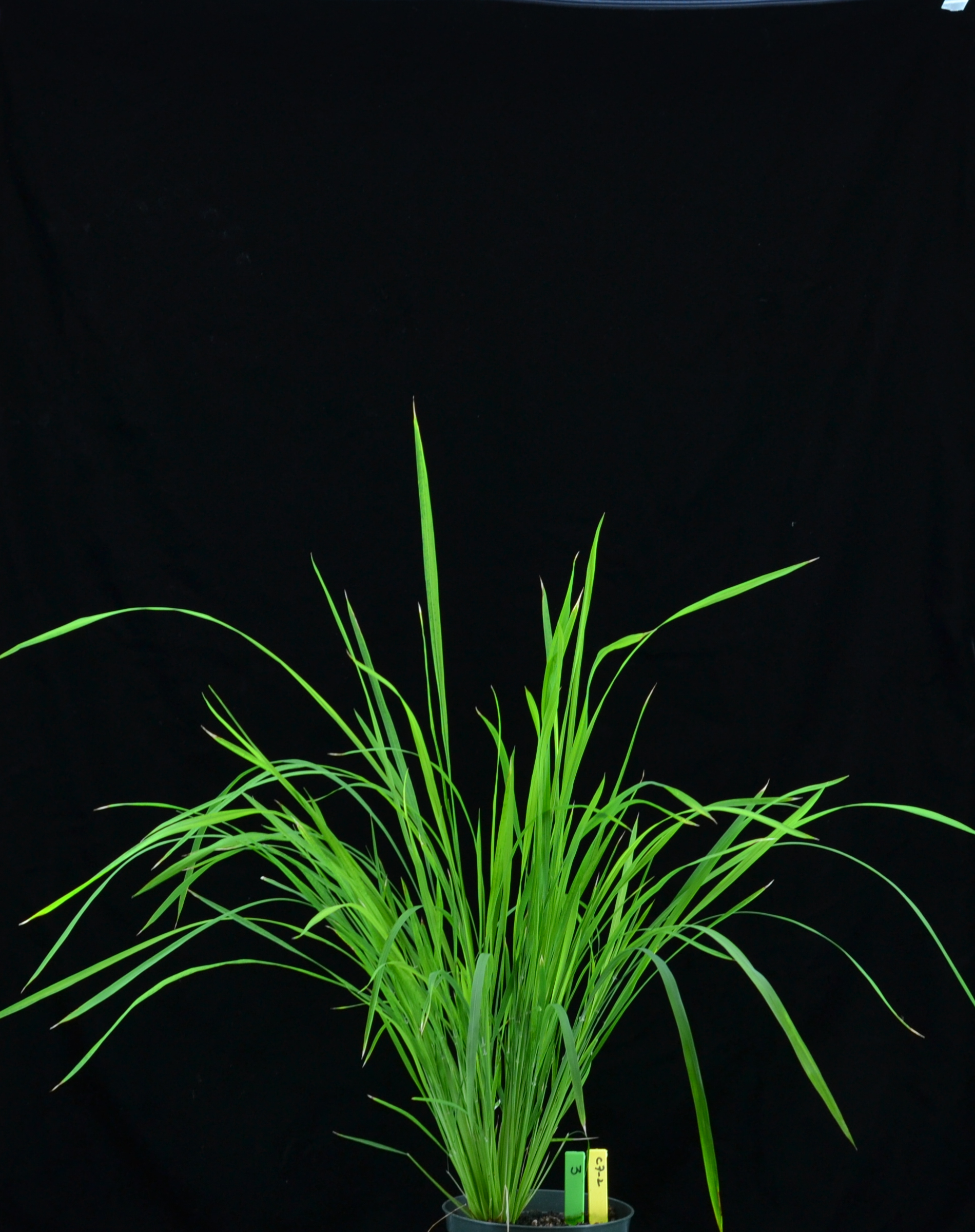

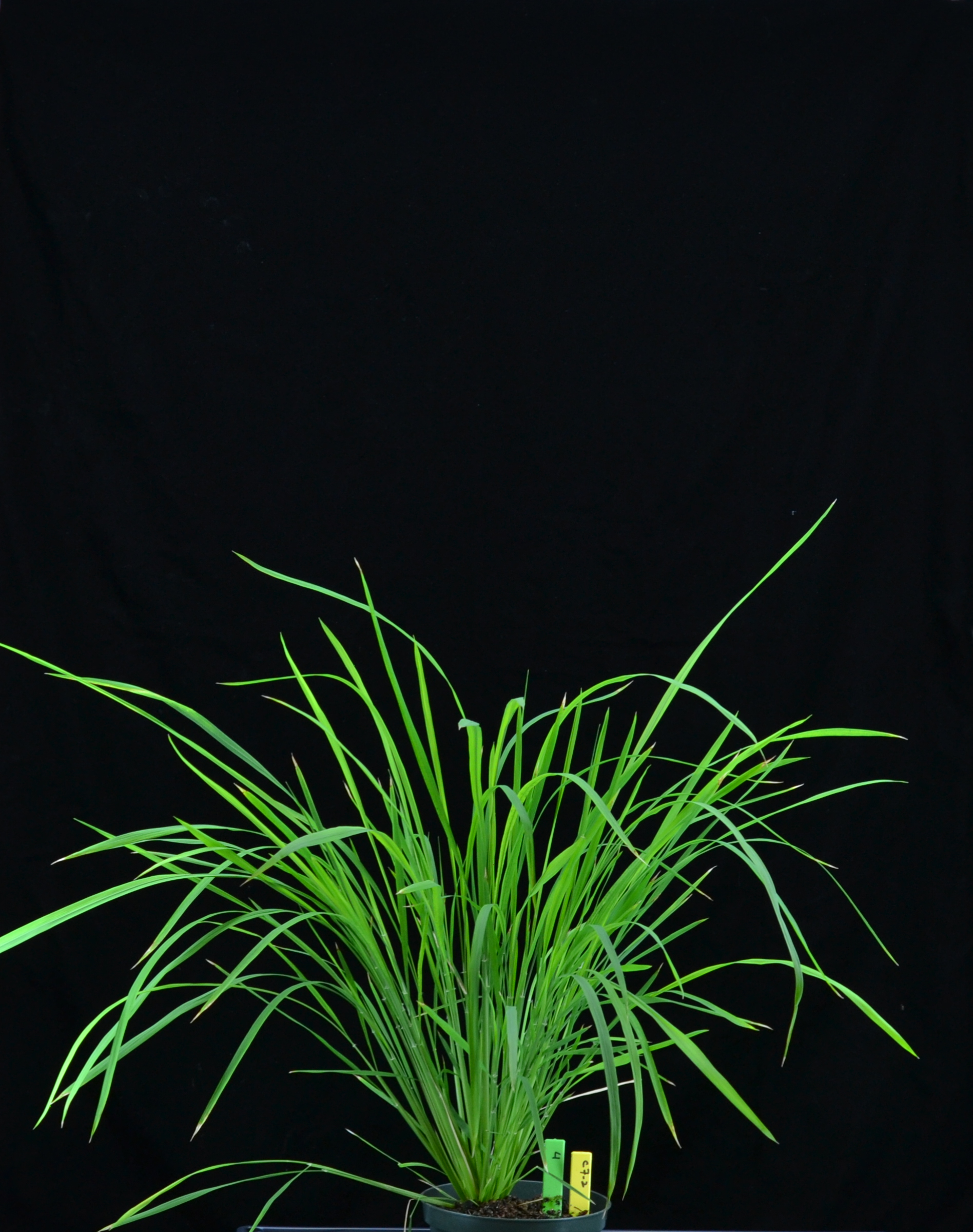


**Control**


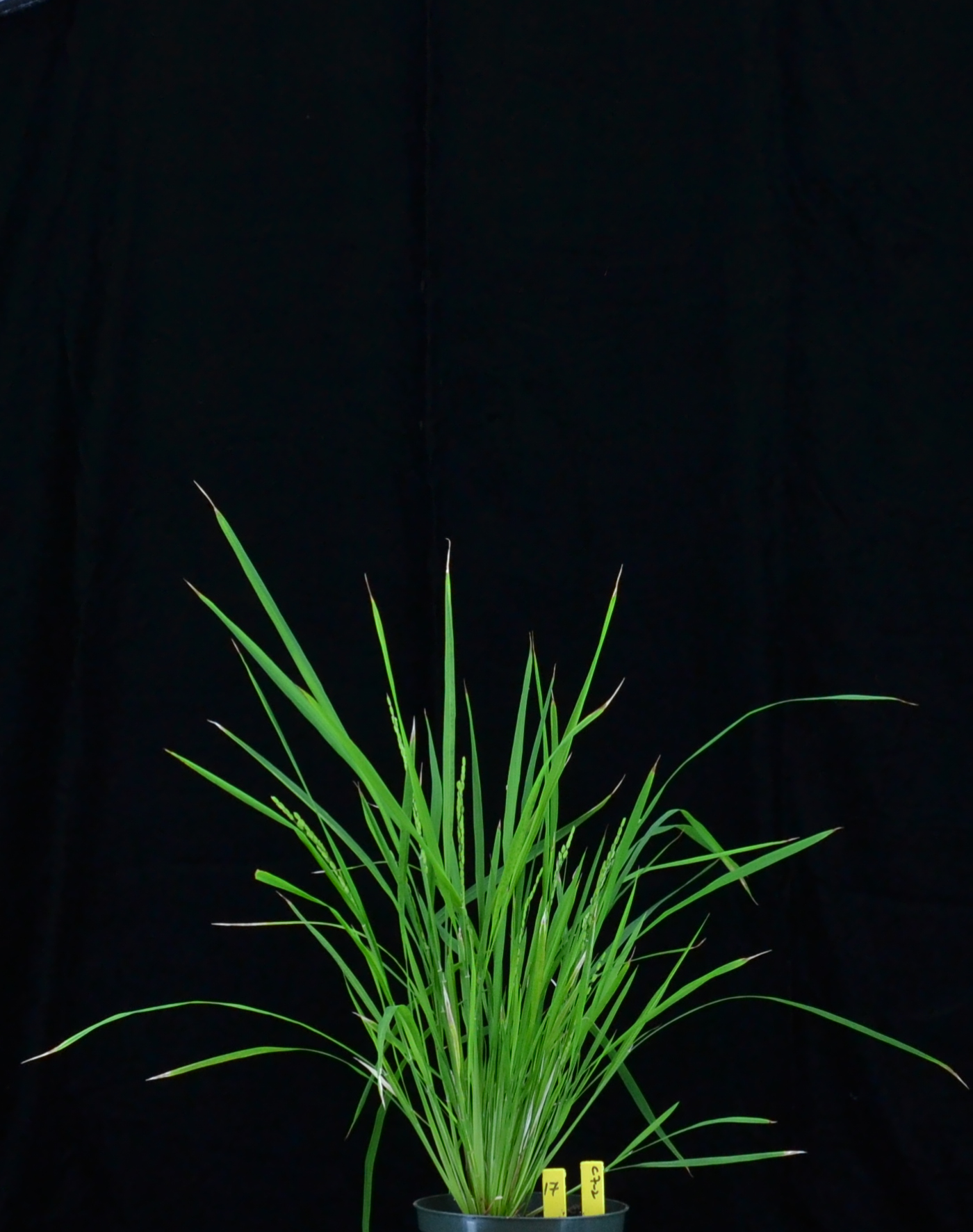

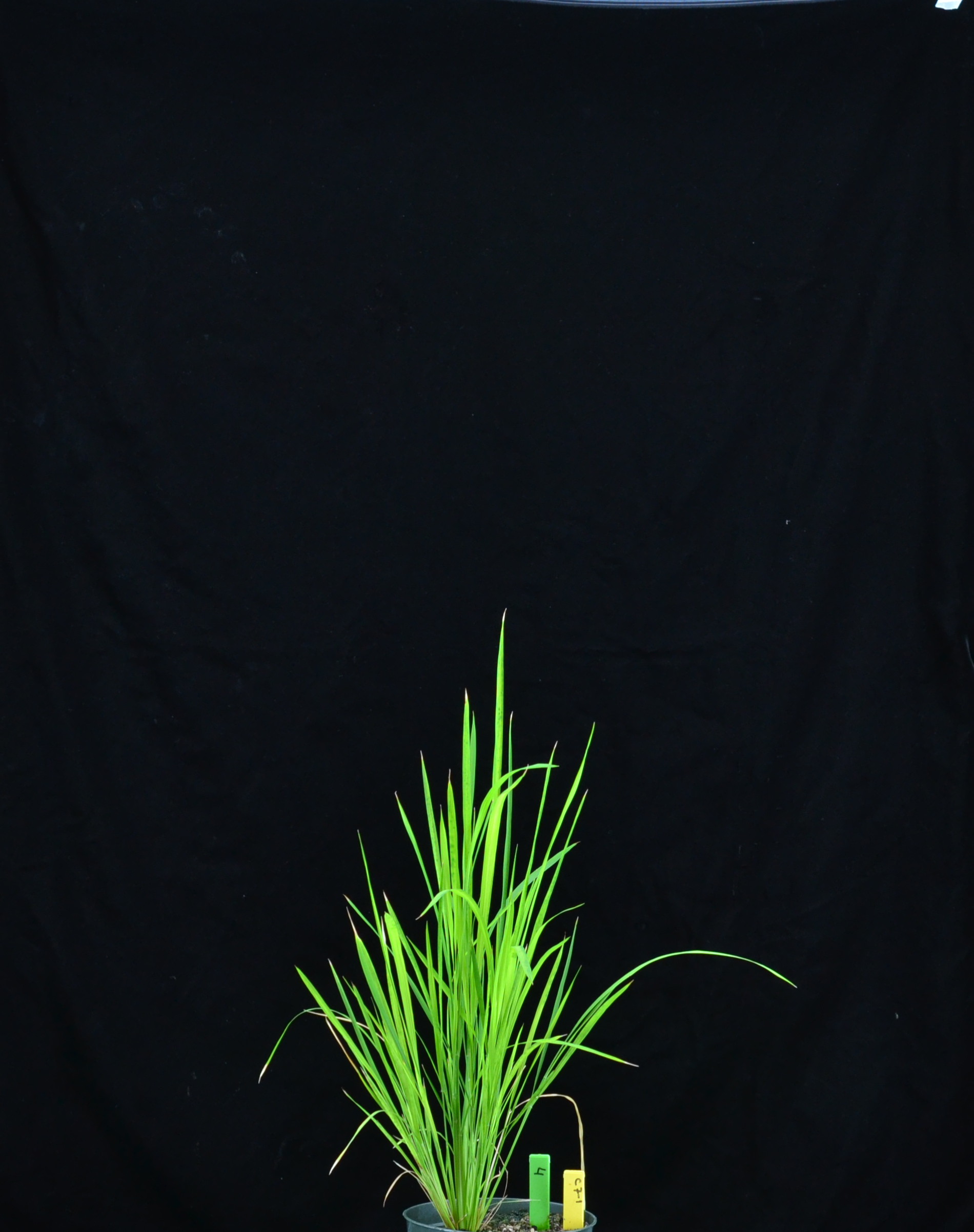


***hl1***

***dl1***

***dl2***

***hl2***

***hl3***

C

**Figure S1: *OsCCD7* targeted mutagenesis: (A)** Genotyping results of T1 generation. For gRNA-2 all of the mutations were bi-allelic. **(B)** All of the mutated plants showed high number of tillers and dwarfism. **(C)** No. of tillers per plant and plant height of each T1 mutant plant was recoreded.

**Fig. S2: CCD7/HTD1 protein variants alignment**

***dl1***

EMBOSS_001 1 MATQAIAPMHAAVVHRHHVLPPRRCVRRRGVFVRASAAAAAAAAETDTLS 50

||||||||||||||||||||||||||||||||||||||||||||||||||

EMBOSS_001 1 MATQAIAPMHAAVVHRHHVLPPRRCVRRRGVFVRASAAAAAAAAETDTLS 50

EMBOSS_001 51 AAFWDYNLLFRSQRDECLDSIPLRVTEGAIPPDFPAGTYYLAGPGIFSDD 100

|||||||||||||||||||||||||||||||||||||||||||. :..

EMBOSS_001 51 AAFWDYNLLFRSQRDECLDSIPLRVTEGAIPPDFPAGTYYLAGR---ASS 97

EMBOSS_001 101 HGSTVHPLDGHGYLRSFRFRPGDRTIHYSARFVETAAKREESRDGASWRF 150

..:|..| ..|...|. .||......|....:....|||.

EMBOSS_001 98 PTTTAPP-----------STPSTATA-TSAPSASGPATAPSTTPRGSWRR 135

EMBOSS_001 151 THRG--PFSVLQGGKKVGNVKVMKNVANTSVLRW---------------G 183

..|| ..:..:||.:.|.......... || |

EMBOSS_001 136 RRRGRRAGTARRGGSRTGGPSPCCRAGR----RWAM*****R**RTWPTPACCG 181

EMBOSS_001 184 GRLLCLWEGGQPYEVDPRTLETVGPFDLLGLAAADDNKATNASAA----- 228

|...|...| ....||..|.| .:..:..:..||:

EMBOSS_001 182 GAAGCSASG----RAASRTRLTPG--------RSRPSARSTCSASPPPTT 219

EMBOSS_001 229 --RRPWLQEAGLDAAARLLRPVLSGVFDMPGKRLLAHYKIDPRRGRLLMV 276

:|..||..|.... ||. ...||....|.:

EMBOSS_001 220 TRQRTRLQHDGRGCR----RPA----------------STPPRACCALFL 249

EMBOSS_001 277 A----CNAEDMLLPRSHFTFYEFDAHFDLVQKREFVVPDHLMIHDWAFTD 322

| |.|... |..|.

EMBOSS_001 250 AGCSTCRARGC----------------------------------WRTTR 265

EMBOSS_001 323 THYILLGNRIKLDIPGSLLALTGTHPMIAALAVDPRRQSTPVYLLPRSPE 372

: ..|..:....|.:....:...|...:.: |||.....||..

EMBOSS_001 266 S---TRGGGVC*WSPATPRTCSSRDPTSLSTS------STPTSTSSRSVS 306

EMBOSS_001 373 TEAGGRDWSV--PIEAPSQMWSVHVGNAFEEANRRGGLDVRLHMSSCSYQ 420

:.......|. |...|:...|. .|::....:| |

EMBOSS_001 307 SSCRTTS*STTGPSPTPTTSSSA-TGSSSTSPDR------------C--- 340

EMBOSS_001 421 WFHFHRMFGYNWHHKKLDPSFMNAAKGKEWLPRLVQVAIELDRTGECRRC 470

||.:.| ....:.| ...|.|..|||

EMBOSS_001 341 -----------WH*RAL------TR*SRRW---------PWTREGSRRRC 364

EMBOSS_001 471 SVRRLSDQHARPADFPA--------INPSYANQRNRFVYAGAASGSRRFL 512

:..|...:..|.|...| ..|..:..|:|...|||||.|....

EMBOSS_001 365 TCFRAPRRPRRAAATGACRSRRRRRCGPCTSATRSRRRTAGAASTSGCTC 414

EMBOSS_001 513 PYFPFDSVVKVD--VSDGSARWW----------------------STDGR 538

...|....:... |:.|:.|.| |:.||

EMBOSS_001 415 QAAPTSGSISTGCLVTIGTTRSWTRRS*TRRRERSGCLASFRWPSSSTGR 464

EMBOSS_001 539 KFVGEPVFVPTGGGEDGGYVLLVEYAVSKHRCHLVVLDAKKI-------- 580

: ..||.:.|| |.:.....:..

EMBOSS_001 465 E--------SAGGAQSGG-------------CPISTPGRRTSRR*TQATP 493

EMBOSS_001 581 --GTENALVAKLEVPKN------------LTFPMGFHGFWGDE*------ 610

||.::..|....|.: .|.||...| |...

EMBOSS_001 494 TRGTGSSTPAPRPAPADSSRTSRSTAW*RSTSPMDRRG--GGLPTGASSS 541

***dl2***

EMBOSS_001 1 MATQAIAPMHAAVVHRHHVLPPRRCVRRRGVFVRASAAAAAAAAETDTLS 50

||||||||||||||||||||||||||||||||||||||||||||||||||

EMBOSS_001 1 MATQAIAPMHAAVVHRHHVLPPRRCVRRRGVFVRASAAAAAAAAETDTLS 50

EMBOSS_001 51 AAFWDYNLLFRSQRDECLDSIPLRVTEGAIPPDFPAGTYYLAG------- 93

||||||||||||||||||||||||||||||||||||||||||.

EMBOSS_001 51 AAFWDYNLLFRSQRDECLDSIPLRVTEGAIPPDFPAGTYYLAAGHLLRRP 100

EMBOSS_001 94 ------------PGIFSDDHGSTVHPLDGHGYLRSFR-------FRPGDR 124

|.:.........||| ||:.| ..||.|

EMBOSS_001 101 RLHRPPPRRPRLPPLLPLPARRPHHPL-----LRAVRGDGGEEGGEPGRR 145

EMBOSS_001 125 --TIHYSARFVETAAKREESR-DGASWRFTHR-----GPFSVLQGG---- 162

.:|.....:..|.:.|..: :|...|..|: .|.::..||

EMBOSS_001 146 VVAVHAPGALLRAAGREEGGQCEGDEERGQHQRAAVGRPAALPLGGRPAV 195

EMBOSS_001 163 ----------------------KKVGNVKVMKNVANTSVLRWGGRLLCLW 190

::.|| :.|.:|:.:..|||

EMBOSS_001 196 RG*****PPDARDRRPVRPARPRRRRRQQGN----ERVCSTTAVAAGGR----- 236

EMBOSS_001 191 EGGQPYEVDPRTLET-VGPFDLLGL--------AAADDNKATNASAARRP 231

||.... ..|....|: .|..|..|..|||..|

EMBOSS_001 237 ---------PRRRRAPAAPCS*RGVRHAGQEAAGALQDRPAAGASADGR- 276

EMBOSS_001 232 WLQEAGLDAAA------RLLRPV---------LSGVFDMPGKRLLAHYKI 266

||..|....| |:.||: .:|....|...|..|...

EMBOSS_001 277 -LQRRGHAPPAIPLHFLRVRRPLRPRPEA*VRRAGPPHDPRLGLHRHPLH 325

EMBOSS_001 267 DPR-------------------------RGRLLMVACNAEDMLLP----- 286

.|| ||....|........||

EMBOSS_001 326 PPRQQDQARHPRIAAGIDGHSPDDRGAGRGPEKAVDAGVPASALPGDRGG 375

EMBOSS_001 287 RSHFTFYEFDAHFDLVQKREFVV--------PDHLMIHDWAFTDTHYILL 328

|......:..|..|:|:.|...| |.....|

EMBOSS_001 376 RPRLERADRGAVADVVRARRQRVRGGEPPGRPRRPAAH------------ 413

EMBOSS_001 329 GNRIKLDIP------GSLLALTGTHPMIAALAVDPRRQSTPVYLLPRSPE 372

:||.:| ...|.|. |..|...|..|.:...|...|||

EMBOSS_001 414 ---VKLLLPVVPFPQDVWLQLA---PQEAGPVVHERGEGKGVAASPRS-- 455

EMBOSS_001 373 TEAGG----RDWSVPIEAPSQMWSVHVGNAFEEANRRGGL---------- 408

|| :|..|| |..|| .|...|.:.|||

EMBOSS_001 456 ---GGHRARQDGRVP-EVLSQ-------EAVRSARQAGGLPGDKPKLRQP 494

EMBOSS_001 409 --DVRLHMSSCSYQWFHFHRMFGYNWHHKKLDPSFMNAAKGK----EWLP 452

.|||....... .::.|......:|: .|:.

EMBOSS_001 495 EEPVRLRRRRVRL---------------PQIPPVLPVRQRGEGRRLRWIG 529

EMBOSS_001 453 RLV---------QVAIELDR-------TGECRRCSVRRLSDQHARPADFP 486

.:| :..:..|| ...||.||:: |..|

EMBOSS_001 530 AVVVYRRAQVRRRAGLRPDRRRRGWWLCSSCRVCSLQ---------AQMP 570

EMBOSS_001 487 AINPSYANQRNRFVYAGAASGSRRFLPYFPFDSVVKVDVSDGSARWWSTD 536

:........|:|....|...|::. |:| |:| ..|

EMBOSS_001 571 SSGAGCKEDRDRECTCGKTRGAKE--PHF----------SNG-IPW---- 603

EMBOSS_001 537 GRKFVGEPVFVPTGGGEDGGYVLLVEYAVSKHRCHLVVLDAKKIGTENAL 586

|:|..:

EMBOSS_001 604 ---FLGR*M----------------------------------------- 609

EMBOSS_001 587 VAKLEVPKNLTFPMGFHGFWGDE* 610

EMBOSS_001 610 ------------------------ 609

***hl1***

EMBOSS_001 1 MATQAIAPMHAAVVHRHHVLPPRRCVRRRGVFVRASAAAAAAAAETDTLS 50

||||||||||||||||||||||||||||||||||||||||||||||||||

EMBOSS_001 1 MATQAIAPMHAAVVHRHHVLPPRRCVRRRGVFVRASAAAAAAAAETDTLS 50

EMBOSS_001 51 AAFWDYNLLFRSQRDECLDSIPLRVTEGAIPPDFPAGTYYLAGPGIFSDD 100

||||||||||||||||||||||||||||||||||||||||||||||||||

EMBOSS_001 51 AAFWDYNLLFRSQRDECLDSIPLRVTEGAIPPDFPAGTYYLAGPGIFSDD 100

EMBOSS_001 101 HGSTVHPLDGHGYLRSFRFRPGDRTIHYSARFVETAAKREESRDGASWRF 150

||||||||||||||||||||||||||||||||||||||||||||||||||

EMBOSS_001 101 HGSTVHPLDGHGYLRSFRFRPGDRTIHYSARFVETAAKREESRDGASWRF 150

EMBOSS_001 151 THRGPFSVLQGGKKVGNVKVMKNVANTSVLRWGGRLLCLWEGGQPYEVDP 200

||||||||||||||||||||||||||||||||||||||||||||||||||

EMBOSS_001 151 THRGPFSVLQGGKKVGNVKVMKNVANTSVLRWGGRLLCLWEGGQPYEVDP 200

EMBOSS_001 201 RTLETVGPFDLLGLAAADDNKATNASAARRPWLQEAGLDAAARLLRPVLS 250

||||||||||||||||||||||||||||||||||||||||||||||||||

EMBOSS_001 201 RTLETVGPFDLLGLAAADDNKATNASAARRPWLQEAGLDAAARLLRPVLS 250

EMBOSS_001 251 GVFDMPGKRLLAHYKIDPRRGRLLMVACNAEDMLLPRSHFTFYEFDAHFD 300

||||||||||||||||||||||||||||||||||||||||||||||||||

EMBOSS_001 251 GVFDMPGKRLLAHYKIDPRRGRLLMVACNAEDMLLPRSHFTFYEFDAHFD 300

EMBOSS_001 301 LVQKREFVVPDHLMIHDWAFTDTHYILLGNRIKLDIPGSLLALTGTHPMI 350

||||||||||||||||||||||||||||||||||||||||||||||||||

EMBOSS_001 301 LVQKREFVVPDHLMIHDWAFTDTHYILLGNRIKLDIPGSLLALTGTHPMI 350

EMBOSS_001 351 AALAVDPRRQSTPVYLLPRSPETEAGGRDWSVPIEAPSQMWSVHVGNAFE 400

||||||||||||||||||||||||||||||||||||||||||||||||||

EMBOSS_001 351 AALAVDPRRQSTPVYLLPRSPETEAGGRDWSVPIEAPSQMWSVHVGNAFE 400

EMBOSS_001 401 EANRRGGLDVRLHMSSCSYQWFHFHRMFGYNWHHKKLDPSFMNAAKGKEW 450

||||||||||||||||||||||||||||||||||||||||||||||||||

EMBOSS_001 401 EANRRGGLDVRLHMSSCSYQWFHFHRMFGYNWHHKKLDPSFMNAAKGKEW 450

EMBOSS_001 451 LPRLVQVAIELDRTGECRRCSVRRLSDQHARPADFPAINPSYANQRNRFV 500

||||||||||||||||||||||||||||||||||||||||||||||||||

EMBOSS_001 451 LPRLVQVAIELDRTGECRRCSVRRLSDQHARPADFPAINPSYANQRNRFV 500

EMBOSS_001 501 YAGAASGSRRFLPYFPFDSVVKVDVSDGSARWWSTDGRKFVGEPVFVPTG 550

||||||||||||||||||||||||||||||||||||||||||||||||||

EMBOSS_001 501 YAGAASGSRRFLPYFPFDSVVKVDVSDGSARWWSTDGRKFVGEPVFVPTG 550

EMBOSS_001 551 GGEDGGYVLLVEYAVSKHRCHLVVLDAKKIGTENALVAKLEVPKNLTFPM 600

||||||||||||||||||||||||||||||||||||||||||||||| .

EMBOSS_001 551 GGEDGGYVLLVEYAVSKHRCHLVVLDAKKIGTENALVAKLEVPKNLT--N 598

EMBOSS_001 601 GFHGFWGDE* 610

|...|.|..

EMBOSS_001 599 GIPWFLGR*****- 607

***hl2***

EMBOSS_001 1 MATQAIAPMHAAVVHRHHVLPPRRCVRRRGVFVRASAAAAAAAAETDTLS 50

||||||||||||||||||||||||||||||||||||||||||||||||||

EMBOSS_001 1 MATQAIAPMHAAVVHRHHVLPPRRCVRRRGVFVRASAAAAAAAAETDTLS 50

EMBOSS_001 51 AAFWDYNLLFRSQRDECLDSIPLRVTEGAIPPDFPAGTYYLAGPGIFSDD 100

||||||||||||||||||||||||||||||||||||||||||||||||||

EMBOSS_001 51 AAFWDYNLLFRSQRDECLDSIPLRVTEGAIPPDFPAGTYYLAGPGIFSDD 100

EMBOSS_001 101 HGSTVHPLDGHGYLRSFRFRPGDRTIHYSARFVETAAKREESRDGASWRF 150

||||||||||||||||||||||||||||||||||||||||||||||||||

EMBOSS_001 101 HGSTVHPLDGHGYLRSFRFRPGDRTIHYSARFVETAAKREESRDGASWRF 150

EMBOSS_001 151 THRGPFSVLQGGKKVGNVKVMKNVANTSVLRWGGRLLCLWEGGQPYEVDP 200

||||||||||||||||||||||||||||||||||||||||||||||||||

EMBOSS_001 151 THRGPFSVLQGGKKVGNVKVMKNVANTSVLRWGGRLLCLWEGGQPYEVDP 200

EMBOSS_001 201 RTLETVGPFDLLGLAAADDNKATNASAARRPWLQEAGLDAAARLLRPVLS 250

||||||||||||||||||||||||||||||||||||||||||||||||||

EMBOSS_001 201 RTLETVGPFDLLGLAAADDNKATNASAARRPWLQEAGLDAAARLLRPVLS 250

EMBOSS_001 251 GVFDMPGKRLLAHYKIDPRRGRLLMVACNAEDMLLPRSHFTFYEFDAHFD 300

||||||||||||||||||||||||||||||||||||||||||||||||||

EMBOSS_001 251 GVFDMPGKRLLAHYKIDPRRGRLLMVACNAEDMLLPRSHFTFYEFDAHFD 300

EMBOSS_001 301 LVQKREFVVPDHLMIHDWAFTDTHYILLGNRIKLDIPGSLLALTGTHPMI 350

||||||||||||||||||||||||||||||||||||||||||||||||||

EMBOSS_001 301 LVQKREFVVPDHLMIHDWAFTDTHYILLGNRIKLDIPGSLLALTGTHPMI 350

EMBOSS_001 351 AALAVDPRRQSTPVYLLPRSPETEAGGRDWSVPIEAPSQMWSVHVGNAFE 400

||||||||||||||||||||||||||||||||||||||||||||||||||

EMBOSS_001 351 AALAVDPRRQSTPVYLLPRSPETEAGGRDWSVPIEAPSQMWSVHVGNAFE 400

EMBOSS_001 401 EANRRGGLDVRLHMSSCSYQWFHFHRMFGYNWHHKKLDPSFMNAAKGKEW 450

||||||||||||||||||||||||||||||||||||||||||||||||||

EMBOSS_001 401 EANRRGGLDVRLHMSSCSYQWFHFHRMFGYNWHHKKLDPSFMNAAKGKEW 450

EMBOSS_001 451 LPRLVQVAIELDRTGECRRCSVRRLSDQHARPADFPAINPSYANQRNRFV 500

||||||||||||||||||||||||||||||||||||||||||||||||||

EMBOSS_001 451 LPRLVQVAIELDRTGECRRCSVRRLSDQHARPADFPAINPSYANQRNRFV 500

EMBOSS_001 501 YAGAASGSRRFLPYFPFDSVVKVDVSDGSARWWSTDGRKFVGEPVFVPTG 550

||||||||||||||||||||||||||||||||||||||||||||||||||

EMBOSS_001 501 YAGAASGSRRFLPYFPFDSVVKVDVSDGSARWWSTDGRKFVGEPVFVPTG 550

EMBOSS_001 551 GGEDGGYVLLVEYAVSKHRCHLVVLDAKKIGTENALVAKLEVPKNLTFPM 600

||||||||||||||||||||||||||||||||||||||||||||||.

EMBOSS_001 551 GGEDGGYVLLVEYAVSKHRCHLVVLDAKKIGTENALVAKLEVPKNLN--- 597

EMBOSS_001 601 GFHGFWGDE* 610

|...|.|..

EMBOSS_001 598 GIPWFLGR*****- 606

***hl3***

EMBOSS_001 1 MATQAIAPMHAAVVHRHHVLPPRRCVRRRGVFVRASAAAAAAAAETDTLS 50

||||||||||||||||||||||||||||||||||||||||||||||||||

EMBOSS_001 1 MATQAIAPMHAAVVHRHHVLPPRRCVRRRGVFVRASAAAAAAAAETDTLS 50

EMBOSS_001 51 AAFWDYNLLFRSQRDECLDSIPLRVTEGAIPPDFPAGTYYLAGPGIFSDD 100

||||||||||||||||||||||||||||||||||||||||||||||||||

EMBOSS_001 51 AAFWDYNLLFRSQRDECLDSIPLRVTEGAIPPDFPAGTYYLAGPGIFSDD 100

EMBOSS_001 101 HGSTVHPLDGHGYLRSFRFRPGDRTIHYSARFVETAAKREESRDGASWRF 150

||||||||||||||||||||||||||||||||||||||||||||||||||

EMBOSS_001 101 HGSTVHPLDGHGYLRSFRFRPGDRTIHYSARFVETAAKREESRDGASWRF 150

EMBOSS_001 151 THRGPFSVLQGGKKVGNVKVMKNVANTSVLRWGGRLLCLWEGGQPYEVDP 200

||||||||||||||||||||||||||||||||||||||||||||||||||

EMBOSS_001 151 THRGPFSVLQGGKKVGNVKVMKNVANTSVLRWGGRLLCLWEGGQPYEVDP 200

EMBOSS_001 201 RTLETVGPFDLLGLAAADDNKATNASAARRPWLQEAGLDAAARLLRPVLS 250

||||||||||||||||||||||||||||||||||||||||||||||||||

EMBOSS_001 201 RTLETVGPFDLLGLAAADDNKATNASAARRPWLQEAGLDAAARLLRPVLS 250

EMBOSS_001 251 GVFDMPGKRLLAHYKIDPRRGRLLMVACNAEDMLLPRSHFTFYEFDAHFD 300

||||||||||||||||||||||||||||||||||||||||||||||||||

EMBOSS_001 251 GVFDMPGKRLLAHYKIDPRRGRLLMVACNAEDMLLPRSHFTFYEFDAHFD 300

EMBOSS_001 301 LVQKREFVVPDHLMIHDWAFTDTHYILLGNRIKLDIPGSLLALTGTHPMI 350

||||||||||||||||||||||||||||||||||||||||||||||||||

EMBOSS_001 301 LVQKREFVVPDHLMIHDWAFTDTHYILLGNRIKLDIPGSLLALTGTHPMI 350

EMBOSS_001 351 AALAVDPRRQSTPVYLLPRSPETEAGGRDWSVPIEAPSQMWSVHVGNAFE 400

||||||||||||||||||||||||||||||||||||||||||||||||||

EMBOSS_001 351 AALAVDPRRQSTPVYLLPRSPETEAGGRDWSVPIEAPSQMWSVHVGNAFE 400

EMBOSS_001 401 EANRRGGLDVRLHMSSCSYQWFHFHRMFGYNWHHKKLDPSFMNAAKGKEW 450

||||||||||||||||||||||||||||||||||||||||||||||||||

EMBOSS_001 401 EANRRGGLDVRLHMSSCSYQWFHFHRMFGYNWHHKKLDPSFMNAAKGKEW 450

EMBOSS_001 451 LPRLVQVAIELDRTGECRRCSVRRLSDQHARPADFPAINPSYANQRNRFV 500

||||||||||||||||||||||||||||||||||||||||||||||||||

EMBOSS_001 451 LPRLVQVAIELDRTGECRRCSVRRLSDQHARPADFPAINPSYANQRNRFV 500

EMBOSS_001 501 YAGAASGSRRFLPYFPFDSVVKVDVSDGSARWWSTDGRKFVGEPVFVPTG 550

||||||||||||||||||||||||||||||||||||||||||||||||||

EMBOSS_001 501 YAGAASGSRRFLPYFPFDSVVKVDVSDGSARWWSTDGRKFVGEPVFVPTG 550

EMBOSS_001 551 GGEDGGYVLLVEYAVSKHRCHLVVLDAKKIGTENALVAKLEVPKNLTFPM 600

|||||||||||||||||||||||||||||||||||||||||||||||||.

EMBOSS_001 551 GGEDGGYVLLVEYAVSKHRCHLVVLDAKKIGTENALVAKLEVPKNLTFPN 600

EMBOSS_001 601 GFHGFWGDE* 610

|...|.|..

EMBOSS_001 601 GIPWFLGR*****- 609

***hl4***

EMBOSS_001 1 MATQAIAPMHAAVVHRHHVLPPRRCVRRRGVFVRASAAAAAAAAETDTLS 50

||||||||||||||||||||||||||||||||||||||||||||||||||

EMBOSS_001 1 MATQAIAPMHAAVVHRHHVLPPRRCVRRRGVFVRASAAAAAAAAETDTLS 50

EMBOSS_001 51 AAFWDYNLLFRSQRDECLDSIPLRVTEGAIPPDFPAGTYYLAGPGIFSDD 100

||||||||||||||||||||||||||||||||||||||||||||||||||

EMBOSS_001 51 AAFWDYNLLFRSQRDECLDSIPLRVTEGAIPPDFPAGTYYLAGPGIFSDD 100

EMBOSS_001 101 HGSTVHPLDGHGYLRSFRFRPGDRTIHYSARFVETAAKREESRDGASWRF 150

||||||||||||||||||||||||||||||||||||||||||||||||||

EMBOSS_001 101 HGSTVHPLDGHGYLRSFRFRPGDRTIHYSARFVETAAKREESRDGASWRF 150

EMBOSS_001 151 THRGPFSVLQGGKKVGNVKVMKNVANTSVLRWGGRLLCLWEGGQPYEVDP 200

||||||||||||||||||||||||||||||||||||||||||||||||||

EMBOSS_001 151 THRGPFSVLQGGKKVGNVKVMKNVANTSVLRWGGRLLCLWEGGQPYEVDP 200

EMBOSS_001 201 RTLETVGPFDLLGLAAADDNKATNASAARRPWLQEAGLDAAARLLRPVLS 250

||||||||||||||||||||||||||||||||||||||||||||||||||

EMBOSS_001 201 RTLETVGPFDLLGLAAADDNKATNASAARRPWLQEAGLDAAARLLRPVLS 250

EMBOSS_001 251 GVFDMPGKRLLAHYKIDPRRGRLLMVACNAEDMLLPRSHFTFYEFDAHFD 300

||||||||||||||||||||||||||||||||||||||||||||||||||

EMBOSS_001 251 GVFDMPGKRLLAHYKIDPRRGRLLMVACNAEDMLLPRSHFTFYEFDAHFD 300

EMBOSS_001 301 LVQKREFVVPDHLMIHDWAFTDTHYILLGNRIKLDIPGSLLALTGTHPMI 350

||||||||||||||||||||||||||||||||||||||||||||||||||

EMBOSS_001 301 LVQKREFVVPDHLMIHDWAFTDTHYILLGNRIKLDIPGSLLALTGTHPMI 350

EMBOSS_001 351 AALAVDPRRQSTPVYLLPRSPETEAGGRDWSVPIEAPSQMWSVHVGNAFE 400

||||||||||||||||||||||||||||||||||||||||||||||||||

EMBOSS_001 351 AALAVDPRRQSTPVYLLPRSPETEAGGRDWSVPIEAPSQMWSVHVGNAFE 400

EMBOSS_001 401 EANRRGGLDVRLHMSSCSYQWFHFHRMFGYNWHHKKLDPSFMNAAKGKEW 450

||||||||||||||||||||||||||||||||||||||||||||||||||

EMBOSS_001 401 EANRRGGLDVRLHMSSCSYQWFHFHRMFGYNWHHKKLDPSFMNAAKGKEW 450

EMBOSS_001 451 LPRLVQVAIELDRTGECRRCSVRRLSDQHARPADFPAINPSYANQRNRFV 500

||||||||||||||||||||||||||||||||||||||||||||||||||

EMBOSS_001 451 LPRLVQVAIELDRTGECRRCSVRRLSDQHARPADFPAINPSYANQRNRFV 500

EMBOSS_001 501 YAGAASGSRRFLPYFPFDSVVKVDVSDGSARWWSTDGRKFVGEPVFVPTG 550

||||||||||||||||||||||||||||||||||||||||||||||||||

EMBOSS_001 501 YAGAASGSRRFLPYFPFDSVVKVDVSDGSARWWSTDGRKFVGEPVFVPTG 550

EMBOSS_001 551 GGEDGGYVLLVEYAVSKHRCHLVVLDAKKIGTENALVAKLEVPKNLT--- 597

|||||||||||||||||||||||||||||||||||||||||||||||

EMBOSS_001 551 GGEDGGYVLLVEYAVSKHRCHLVVLDAKKIGTENALVAKLEVPKNLT*****WD 600

EMBOSS_001 598 ----FPMGFHGFWGDE* 610

..|.

EMBOSS_001 601 SMVSGEMN--------- 608

***hl5***

EMBOSS_001 1 MATQAIAPMHAAVVHRHHVLPPRRCVRRRGVFVRASAAAAAAAAETDTLS 50

||||||||||||||||||||||||||||||||||||||||||||||||||

EMBOSS_001 1 MATQAIAPMHAAVVHRHHVLPPRRCVRRRGVFVRASAAAAAAAAETDTLS 50

EMBOSS_001 51 AAFWDYNLLFRSQRDECLDSIPLRVTEGAIPPDFPAGTYYLAGPGIFSDD 100

||||||||||||||||||||||||||||||||||||||||||||||||||

EMBOSS_001 51 AAFWDYNLLFRSQRDECLDSIPLRVTEGAIPPDFPAGTYYLAGPGIFSDD 100

EMBOSS_001 101 HGSTVHPLDGHGYLRSFRFRPGDRTIHYSARFVETAAKREESRDGASWRF 150

||||||||||||||||||||||||||||||||||||||||||||||||||

EMBOSS_001 101 HGSTVHPLDGHGYLRSFRFRPGDRTIHYSARFVETAAKREESRDGASWRF 150

EMBOSS_001 151 THRGPFSVLQGGKKVGNVKVMKNVANTSVLRWGGRLLCLWEGGQPYEVDP 200

||||||||||||||||||||||||||||||||||||||||||||||||||

EMBOSS_001 151 THRGPFSVLQGGKKVGNVKVMKNVANTSVLRWGGRLLCLWEGGQPYEVDP 200

EMBOSS_001 201 RTLETVGPFDLLGLAAADDNKATNASAARRPWLQEAGLDAAARLLRPVLS 250

||||||||||||||||||||||||||||||||||||||||||||||||||

EMBOSS_001 201 RTLETVGPFDLLGLAAADDNKATNASAARRPWLQEAGLDAAARLLRPVLS 250

EMBOSS_001 251 GVFDMPGKRLLAHYKIDPRRGRLLMVACNAEDMLLPRSHFTFYEFDAHFD 300

||||||||||||||||||||||||||||||||||||||||||||||||||

EMBOSS_001 251 GVFDMPGKRLLAHYKIDPRRGRLLMVACNAEDMLLPRSHFTFYEFDAHFD 300

EMBOSS_001 301 LVQKREFVVPDHLMIHDWAFTDTHYILLGNRIKLDIPGSLLALTGTHPMI 350

||||||||||||||||||||||||||||||||||||||||||||||||||

EMBOSS_001 301 LVQKREFVVPDHLMIHDWAFTDTHYILLGNRIKLDIPGSLLALTGTHPMI 350

EMBOSS_001 351 AALAVDPRRQSTPVYLLPRSPETEAGGRDWSVPIEAPSQMWSVHVGNAFE 400

||||||||||||||||||||||||||||||||||||||||||||||||||

EMBOSS_001 351 AALAVDPRRQSTPVYLLPRSPETEAGGRDWSVPIEAPSQMWSVHVGNAFE 400

EMBOSS_001 401 EANRRGGLDVRLHMSSCSYQWFHFHRMFGYNWHHKKLDPSFMNAAKGKEW 450

||||||||||||||||||||||||||||||||||||||||||||||||||

EMBOSS_001 401 EANRRGGLDVRLHMSSCSYQWFHFHRMFGYNWHHKKLDPSFMNAAKGKEW 450

EMBOSS_001 451 LPRLVQVAIELDRTGECRRCSVRRLSDQHARPADFPAINPSYANQRNRFV 500

||||||||||||||||||||||||||||||||||||||||||||||||||

EMBOSS_001 451 LPRLVQVAIELDRTGECRRCSVRRLSDQHARPADFPAINPSYANQRNRFV 500

EMBOSS_001 501 YAGAASGSRRFLPYFPFDSVVKVDVSDGSARWWSTDGRKFVGEPVFVPTG 550

||||||||||||||||||||||||||||||||||||||||||||||||||

EMBOSS_001 501 YAGAASGSRRFLPYFPFDSVVKVDVSDGSARWWSTDGRKFVGEPVFVPTG 550

EMBOSS_001 551 GGEDGGYVLLVEYAVSKHRCHLVVLDAKKIGTENALVAKLEVPKNLTFPM 600

||||||||||||||||||||||||||||||||||||||||||||||||.

EMBOSS_001 551 GGEDGGYVLLVEYAVSKHRCHLVVLDAKKIGTENALVAKLEVPKNLTFQ- 599

EMBOSS_001 601 GFHGFWGDE*---------------------------------------- 610

|....

EMBOSS_001 600 -----WDSMVSGEMNEHRASIRSSLTLERNCLEKARILPRVLIKESFVII 644

EMBOSS_001 611 ------------- 610

EMBOSS_001 645 CTWWRGIIRGTK***** 657

***hl6***

EMBOSS_001 1 MATQAIAPMHAAVVHRHHVLPPRRCVRRRGVFVRASAAAAAAAAETDTLS 50

||||||||||||||||||||||||||||||||||||||||||||||||||

EMBOSS_001 1 MATQAIAPMHAAVVHRHHVLPPRRCVRRRGVFVRASAAAAAAAAETDTLS 50

EMBOSS_001 51 AAFWDYNLLFRSQRDECLDSIPLRVTEGAIPPDFPAGTYYLAGPGIFSDD 100

||||||||||||||||||||||||||||||||||||||||||||||||||

EMBOSS_001 51 AAFWDYNLLFRSQRDECLDSIPLRVTEGAIPPDFPAGTYYLAGPGIFSDD 100

EMBOSS_001 101 HGSTVHPLDGHGYLRSFRFRPGDRTIHYSARFVETAAKREESRDGASWRF 150

||||||||||||||||||||||||||||||||||||||||||||||||||

EMBOSS_001 101 HGSTVHPLDGHGYLRSFRFRPGDRTIHYSARFVETAAKREESRDGASWRF 150

EMBOSS_001 151 THRGPFSVLQGGKKVGNVKVMKNVANTSVLRWGGRLLCLWEGGQPYEVDP 200

||||||||||||||||||||||||||||||||||||||||||||||||||

EMBOSS_001 151 THRGPFSVLQGGKKVGNVKVMKNVANTSVLRWGGRLLCLWEGGQPYEVDP 200

EMBOSS_001 201 RTLETVGPFDLLGLAAADDNKATNASAARRPWLQEAGLDAAARLLRPVLS 250

||||||||||||||||||||||||||||||||||||||||||||||||||

EMBOSS_001 201 RTLETVGPFDLLGLAAADDNKATNASAARRPWLQEAGLDAAARLLRPVLS 250

EMBOSS_001 251 GVFDMPGKRLLAHYKIDPRRGRLLMVACNAEDMLLPRSHFTFYEFDAHFD 300

||||||||||||||||||||||||||||||||||||||||||||||||||

EMBOSS_001 251 GVFDMPGKRLLAHYKIDPRRGRLLMVACNAEDMLLPRSHFTFYEFDAHFD 300

EMBOSS_001 301 LVQKREFVVPDHLMIHDWAFTDTHYILLGNRIKLDIPGSLLALTGTHPMI 350

||||||||||||||||||||||||||||||||||||||||||||||||||

EMBOSS_001 301 LVQKREFVVPDHLMIHDWAFTDTHYILLGNRIKLDIPGSLLALTGTHPMI 350

EMBOSS_001 351 AALAVDPRRQSTPVYLLPRSPETEAGGRDWSVPIEAPSQMWSVHVGNAFE 400

||||||||||||||||||||||||||||||||||||||||||||||||||

EMBOSS_001 351 AALAVDPRRQSTPVYLLPRSPETEAGGRDWSVPIEAPSQMWSVHVGNAFE 400

EMBOSS_001 401 EANRRGGLDVRLHMSSCSYQWFHFHRMFGYNWHHKKLDPSFMNAAKGKEW 450

||||||||||||||||||||||||||||||||||||||||||||||||||

EMBOSS_001 401 EANRRGGLDVRLHMSSCSYQWFHFHRMFGYNWHHKKLDPSFMNAAKGKEW 450

EMBOSS_001 451 LPRLVQVAIELDRTGECRRCSVRRLSDQHARPADFPAINPSYANQRNRFV 500

||||||||||||||||||||||||||||||||||||||||||||||||||

EMBOSS_001 451 LPRLVQVAIELDRTGECRRCSVRRLSDQHARPADFPAINPSYANQRNRFV 500

EMBOSS_001 501 YAGAASGSRRFLPYFPFDSVVKVDVSDGSARWWSTDGRKFVGEPVFVPTG 550

||||||||||||||||||||||||||||||||||||||||||||||||||

EMBOSS_001 501 YAGAASGSRRFLPYFPFDSVVKVDVSDGSARWWSTDGRKFVGEPVFVPTG 550

EMBOSS_001 551 GGEDGGYVLLVEYAVSKHRCHLVVLDAKKIGTENALVAKLEVPKNLTFPM 600

||||||||||||||||||||||||||||||||||||||||||||||||

EMBOSS_001 551 GGEDGGYVLLVEYAVSKHRCHLVVLDAKKIGTENALVAKLEVPKNLTF-- 598

EMBOSS_001 601 GFHGFWGDE*- 610

|...|.| .

EMBOSS_001 599 GIPWFLG-R*****M 608

***hl7***

EMBOSS_001 1 MATQAIAPMHAAVVHRHHVLPPRRCVRRRGVFVRASAAAAAAAAETDTLS 50

||||||||||||||||||||||||||||||||||||||||||||||||||

EMBOSS_001 1 MATQAIAPMHAAVVHRHHVLPPRRCVRRRGVFVRASAAAAAAAAETDTLS 50

EMBOSS_001 51 AAFWDYNLLFRSQRDECLDSIPLRVTEGAIPPDFPAGTYYLAGPGIFSDD 100

||||||||||||||||||||||||||||||||||||||||||||||||||

EMBOSS_001 51 AAFWDYNLLFRSQRDECLDSIPLRVTEGAIPPDFPAGTYYLAGPGIFSDD 100

EMBOSS_001 101 HGSTVHPLDGHGYLRSFRFRPGDRTIHYSARFVETAAKREESRDGASWRF 150

||||||||||||||||||||||||||||||||||||||||||||||||||

EMBOSS_001 101 HGSTVHPLDGHGYLRSFRFRPGDRTIHYSARFVETAAKREESRDGASWRF 150

EMBOSS_001 151 THRGPFSVLQGGKKVGNVKVMKNVANTSVLRWGGRLLCLWEGGQPYEVDP 200

||||||||||||||||||||||||||||||||||||||||||||||||||

EMBOSS_001 151 THRGPFSVLQGGKKVGNVKVMKNVANTSVLRWGGRLLCLWEGGQPYEVDP 200

EMBOSS_001 201 RTLETVGPFDLLGLAAADDNKATNASAARRPWLQEAGLDAAARLLRPVLS 250

||||||||||||||||||||||||||||||||||||||||||||||||||

EMBOSS_001 201 RTLETVGPFDLLGLAAADDNKATNASAARRPWLQEAGLDAAARLLRPVLS 250

EMBOSS_001 251 GVFDMPGKRLLAHYKIDPRRGRLLMVACNAEDMLLPRSHFTFYEFDAHFD 300

||||||||||||||||||||||||||||||||||||||||||||||||||

EMBOSS_001 251 GVFDMPGKRLLAHYKIDPRRGRLLMVACNAEDMLLPRSHFTFYEFDAHFD 300

EMBOSS_001 301 LVQKREFVVPDHLMIHDWAFTDTHYILLGNRIKLDIPGSLLALTGTHPMI 350

||||||||||||||||||||||||||||||||||||||||||||||||||

EMBOSS_001 301 LVQKREFVVPDHLMIHDWAFTDTHYILLGNRIKLDIPGSLLALTGTHPMI 350

EMBOSS_001 351 AALAVDPRRQSTPVYLLPRSPETEAGGRDWSVPIEAPSQMWSVHVGNAFE 400

||||||||||||||||||||||||||||||||||||||||||||||||||

EMBOSS_001 351 AALAVDPRRQSTPVYLLPRSPETEAGGRDWSVPIEAPSQMWSVHVGNAFE 400

EMBOSS_001 401 EANRRGGLDVRLHMSSCSYQWFHFHRMFGYNWHHKKLDPSFMNAAKGKEW 450

||||||||||||||||||||||||||||||||||||||||||||||||||

EMBOSS_001 401 EANRRGGLDVRLHMSSCSYQWFHFHRMFGYNWHHKKLDPSFMNAAKGKEW 450

EMBOSS_001 451 LPRLVQVAIELDRTGECRRCSVRRLSDQHARPADFPAINPSYANQRNRFV 500

||||||||||||||||||||||||||||||||||||||||||||||||||

EMBOSS_001 451 LPRLVQVAIELDRTGECRRCSVRRLSDQHARPADFPAINPSYANQRNRFV 500

EMBOSS_001 501 YAGAASGSRRFLPYFPFDSVVKVDVSDGSARWWSTDGRKFVGEPVFVPTG 550

||||||||||||||||||||||||||||||||||||||||||||||||||

EMBOSS_001 501 YAGAASGSRRFLPYFPFDSVVKVDVSDGSARWWSTDGRKFVGEPVFVPTG 550

EMBOSS_001 551 GGEDGGYVLLVEYAVSKHRCHLVVLDAKKIGTENALVAKLEVPKNLTFPM 600

||||||||||||||||||||||||||||||||||||||||||||||| .

EMBOSS_001 551 GGEDGGYVLLVEYAVSKHRCHLVVLDAKKIGTENALVAKLEVPKNLT--N 598

EMBOSS_001 601 GFHGFWGDE*- 610

|...|.| .

EMBOSS_001 599 GIPWFLG-R*****M 608

**Table S1: List of Primers used in this study**

| Primer name | Sequence | Purpose |
| --- | --- | --- |
| F1 | GATGGCAACACAAGCGATTG | Genotyping of CCD7-gRNA-1 target |
| R1 | GTTCGTTGCCTTGTTGTCGTC | Genotyping of CCD7-gRNA-1 target |
| F3 | CTTCTGGGACTACAACCTCC | Genotyping of CCD7-gRNA-1 target |
| R3 | CACGTTCTTCATCACCTTCAC | Genotyping of CCD7-gRNA-1 target |
| F5 | CAGATTCCTCCCGTACTTCCC | Genotyping of CCD7-gRNA-2 target |
| R5 | GACTGGATGTACAGTAGTTATTTGG | Genotyping of CCD7-gRNA-2 target |

**Table S2: Sequence of gRNAs**

| **gRNA/Repair template** | **5'-Sequence-3'** |
| --- | --- |
| U3 promoter | GACCATGATTACGCCAAGCTTAAGGAATCTTTAAACATACGAACAGATCACTTAAAGTTCTTCTGAAGCAACTTAAAGTTATCAGGCATGCATGGATCTTGGAGGAATCAGATGTGCAGTCAGGGACCATAGCACAAGACAGGCGTCTTCTACTGGTGCTACCAGCAAATGCTGGAAGCCGGGAACACTGGGTACGTTGGAAACCACGTGATGTGAAGAAGTAAGATAAACTGTAGGAGAAAAGCATTTCGTAGTGGGCCATGAAGCCTTTCAGGACATGTATTGCAGTATGGGCCGGCCCATTACGCAATTGGACGACAACAAAGACTAGTATTAGTACCACCTCGGCTATCCACATAGATCAAAGCTGATTTAAAAGAGTTGTGCAGATGATCCGTGGCA |
| CCD7_gRNA-1 | AACAAAGCACCAGTGGTCTAGTGGTAGAATAGTACCCTGCCACGGTACAGACCCGGGTTCGATTCCCGGCTGGTGCAACCTACTACCTCGCCGGGCCGTTTTAGAGCTAGAAATAGCAAGTTAAAATAAGGCTAGTCCGTTATCAACTTGAAAAAGTGGCACCGAGTCGGTGCTTTTTTTTTT |
| CCD7_gRNA-1 | AACAAAGCACCAGTGGTCTAGTGGTAGAATAGTACCCTGCCACGGTACAGACCCGGGTTCGATTCCCGGCTGGTGCAAAGAACCTCACTTTTCCAATGTTTTAGAGCTAGAAATAGCAAGTTAAAATAAGGCTAGTCCGTTATCAACTTGAAAAAGTGGCACCGAGTCGGTGCTTTTTTTTTT |

pre-tRNA: green background gRNA spacer: blue background

Scaffold RNA: yellow background polIII terminator: red background
